# A simple route for renewable nano-sized arjunolic and asiatic acids and self-assembly of arjuna-bromolactone

**DOI:** 10.3762/bjoc.4.24

**Published:** 2008-07-09

**Authors:** Braja G Bag, Partha P Dey, Shaishab K Dinda, William S Sheldrick, Iris M Oppel

**Affiliations:** 1 Department of Chemistry and Chemical Technology, Vidyasagar University, Midnapore 721 102, India; 2 Lehrstuhl fuer Analytische Chemie, Ruhr University, D 44780 Bochum, Germany

**Keywords:** arjunolic acid, nanochemistry, renewable, self-assembly, triterpene

## Abstract

While separating two natural nano-sized triterpenic acids via bromolactonization, we serendipitously discovered that arjuna-bromolactone is an excellent gelator of various organic solvents. A simple and efficient method for the separation of two triterpenic acids and the gelation ability and solid state 1D-helical self-assembly of nano-sized arjuna-bromolactone are reported.

## Introduction

Triterpenes are an important class of plant secondary metabolites derived from C30 precursors [1–2]. More than 100 triterpenoids with different skeletons and functional groups have been isolated and structurally characterized. These may not be essential for the life of the plant, but play an important role in self-defence against harmful organisms and coloring petals and fruits etc., and medicinal uses of these materials are known [3–4]. Recently, triterpenoids have been recognized as *renewables* in supramolecular chemistry and nanoscience [5–10]. Even though the nano-sized triterpenic acids are available in abundance from a variety of plants, a major difficulty in their use is their availability in pure form. Occurrence of the pentacyclic triterpenoids having a β-amyrin skeleton with certain amounts of α-amyrins is common in nature and has been explained by 1,2-CH_3_ migration during their biosynthesis [11]. The mixture of the triterpenic acids extractable from *Terminalia arjuna* contains arjunolic acid as the major component along with asiatic acid, as a minor component having a close structural resemblance [12–13]. Biotransformation of the ursane to the oleanane skeleton has recently been reported [14], but no simple method for the separation of the two triterpenic acids is known [15]. Herein we report a simple method for separation the two nano-sized triterpenic acids along with the self-assembly property of arjuna-bromolactone in organic solvents and its 1D-helical structure in the solid state.

## Results and Discussion

**Scheme 1:**
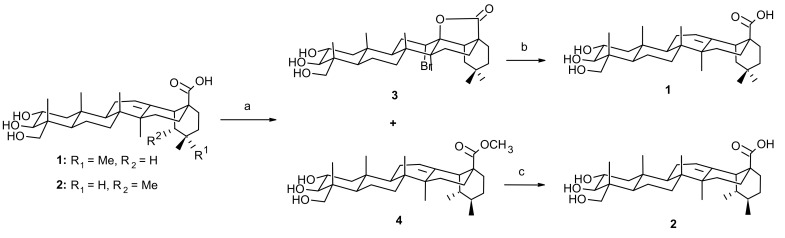
(a) (i) Br_2_/AcOH, (ii) CH_2_N_2_, (iii) separation by crystallization, (b) Zn/AcOH/RT (c) LiBr/DMF. The carbon skeletons of both arjunolic and asiatic acids are 1.15 nm (3C•••21C) long [10].

**Figure 1:**
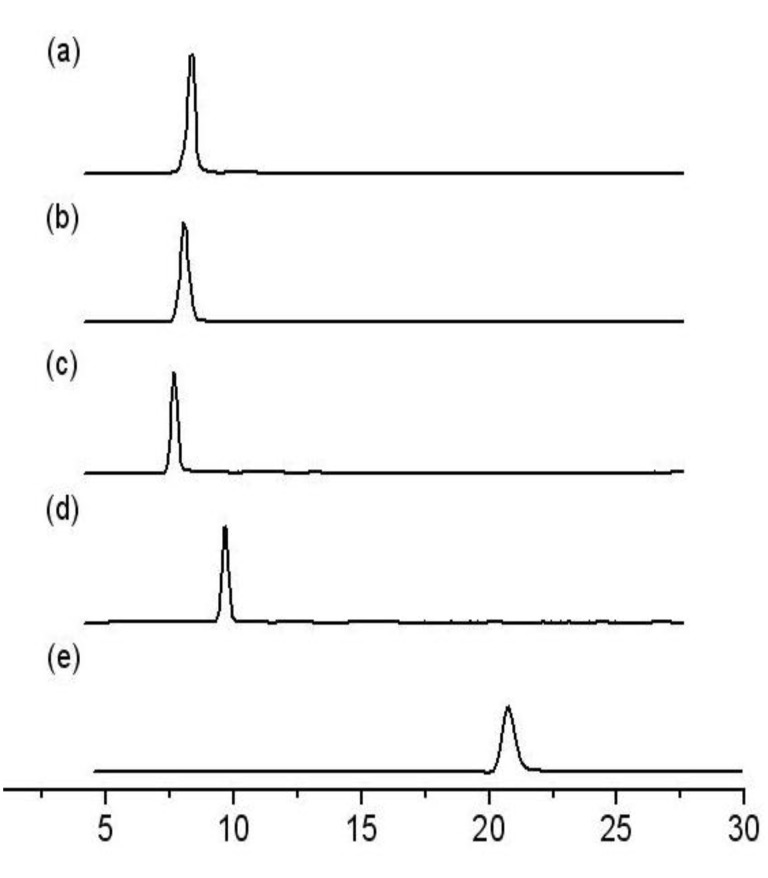
Reversed-phase HPLC analysis. Conditions: C_18_-column 8 mm x 10 cm, mobile phase 6:1 methanol/water (flow rate 0.7 mL/minute), UV-Vis detection at 206 nm. All the triterpenic acid samples were injected after dissolving in methanol/acetic acid mixture to obtain a sharp peak. HPLC profiles: a: *t*_R_ [arjunolic acid (**1**)] = 8.3 min, b: *t*_R_ (mixture of the triterpenic acids **1** and **2**) = 8.7 min, c: *t*_R_ [asiatic acid (**2**)] = 9.0 min), d: *t*_R_ (arjuna-bromolactone **3**) = 10.0 min), e: *t*_R_ [methyl asiatate (**4**)] = 20.7 min.

The mixture of the triterpenic acids **1** and **2** obtained from *Terminalia arjuna* (see Supporting Information File 1) was transformed to a mixture of arjuna-bromolactone **3** and unchanged asiatic acid (**2**) on reaction with bromine in acetic acid, using the reactivity differences of the triterpenic acids towards bromolactonization [9,15]. A suspension of the mixture of **2** and **3** in methanol with ethereal diazomethane yielded a mixture of **3** and **4** (Scheme 1). To our delight, we noticed that, arjuna-bromolactone **3** crystallized out in pure form from a solution of the mixture in ethyl acetate leaving methyl asiatate (**4**) exclusively in the mother liquor. When initiated with 5 g of the mixture of triterpenic acids, 3.6 g of arjuna-bromolactone **3** was isolated in pure form leaving 1.2 g of methyl asiatate (**4**) in the mother liquor (see Figure 1 for HPLC profiles).

Arjuna-bromolactone **3** (3.6 g) on stirring with Zn-dust (20 equivalents) in acetic acid at room temperature for 30 min produced arjunolic acid (3 g) in 97% isolated yield [12]. Hydrolysis of methyl asiatate (1.1 g) by refluxing with LiBr/DMF produced asiatic acid (1 g) in 90% yield [16]. All the transformations were monitored by HPLC (see Figure 1) using a reverse phase analytical column and a UV-Visible detector (at 206 nm). In the ^1^H-NMR spectrum (600 MHz) six singlets were observed [δ 1.12 (s, 3H), 0.91 (s, 3H), 0.87 (s, 6H, 2 CH_3_'s), 0.70 (s, 3H), 0.53 (s, 3H) ppm] in the high field region for six methyl groups of arjunolic acid, supporting β-amyrin type skeleton. In asiatic acid four methyl groups appear as singlets [δ 1.03 (s, 3H), 0.91 (s, 3H), 0.72 (s, 3H), 0.52 (s, 3H) ppm] and two methyl groups appear as doublets [δ 0.905 (d, *J* = 9.0 Hz, 3H), 0.803 (d, *J* = 6.0 Hz, 3H) ppm] in the high field region, supporting assignement of α-amyrin type skeleton. The two triterpenic acids appear as a single peak by reverse phase HPLC (Figure 1b). An 80:20 mixture of arjunolic acid and asiatic acid was established from the HPLC peak areas of the corresponding methyl esters.

While attempting crystallization of arjuna-bromolactone **3** from various solvents we serendipitously discovered that it formed gels efficiently in various aromatic solvents (Table 1) [17–18]. In benzene as the solvent, compound **3** formed a transparent gel that was stable for several weeks. But the transparent gel in mesitylene gave rise to the formation of crystals, with concomitant cleavage of the gel network, on standing at room temperature for about a day.

**Table 1:** Gelation test results^a^.

Entry	solvents	**3**	**1**	**2**

1	benzene	G (0.19)	I	I
2	toluene	PG (0.66)	I	I
3	*o*-xylene	G (0.40)	I	I
4	*m*-xylene	PG (1.0)	I	I
5	*p*-xylene	PG (1.0)	I	I
6	mesitylene	G (0.50)	I	I
7	chlorobenzene	G (5.0)	I	I
8	bromobenzene	G (5.0)	I	I
9	ethyl acetate	C	I	I

^a^G = gel, PG = partial gel, I = insoluble. Minimum gel conc. (g/100 mL) is given in the parenthesis

**Figure 2:**
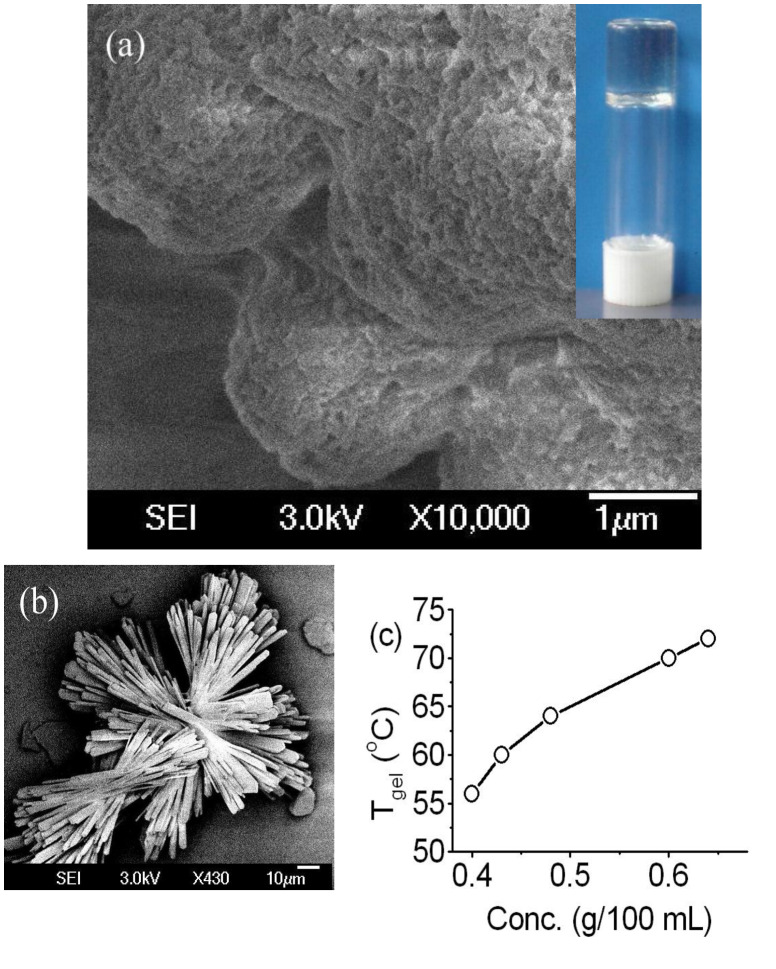
Scanning electron micrographs of the dried gels of compound **3** in mesitylene (a, inset: inverted vial containing a gel in mesitylene) and *o*-xylene (b). (c) Plot of *T*_gel_ vs conc. (g/100 mL) in *o*-xylene.

Scanning electron micrographs of the dried gels revealed a fibrous network structure having fibers of submicron diameters (Figure 2a). With increasing concentration of compound **3**, the gel to sol transition temperature (*T*_gel_) increased indicating an increase in the degree of branching in the fibrillar network (Figure 2c) [17–18].

**Figure 3:**
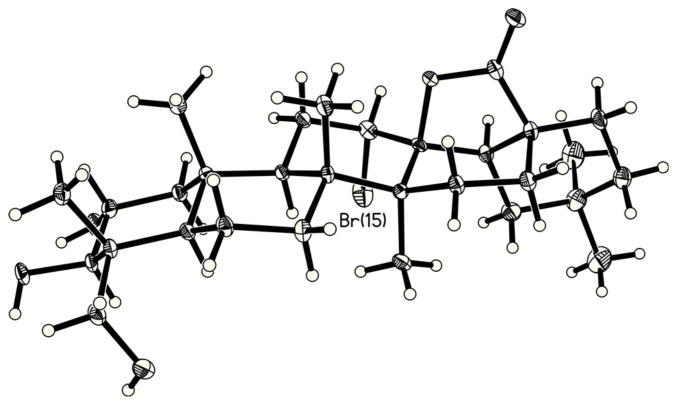
X-ray structure of arjuna-bromolactone **3**. The length of the molecule (3O•••30C) is 1.26 nm.

**Figure 4:**
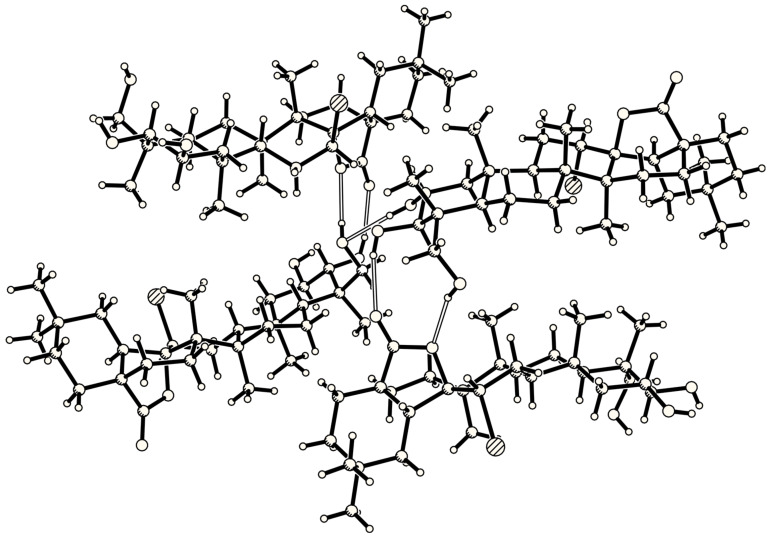
Crystal packing diagram of arjuna-bromolactone **3**. A network of hydrogen bridges leading to the formation of a one-dimensional helix is observed.

An X-ray quality crystal was obtained from ethyl acetate (Figure 3). Within the packing, a network of hydrogen bridges was found leading to the formation of a one-dimensional helix (Figure 4). The observed (OH•••O) distances exhibit values of 2.851(5) and 2.920(5) Å for the double-bridge and 2.886(5) Å for the single one with angles at the H atoms of 157.2(3)°, 167.1(3)° and 152.1(3)° respectively.

## Conclusion

In conclusion, we have developed a simple route to obtain the nano-sized arjunolic and asiatic acids in pure form affording >75% overall isolated yield of the materials. The nano-sized bromolactone **3** formed gels efficiently in various aromatic solvents. Scanning electron micrographs of the dried gels of **3** revealed a fibrous network structure having fibers of submicron diameters. Solid state structure of **3** revealed a 1D-helical self-assembly indicating the specific mode of packing of the molecules within the fibrillar networks [17–18]. We propose that the procedure outlined here to obtain the *renewable* nanosized triterpenic acids in pure form and the self-assembled fibrillar networks obtained in the aromatic solvents will find applications in various facets of supramolecular chemistry and nanoscience [19–20].

## Experimental

**Arjunolic acid **(**1**): Arjuna-bromolactone **3** (see Supporting Information File 1, 3.6 g, 6.35 mmol) was dissolved in glacial acetic acid (105 mL) and the solution was treated with zinc dust (8.34 g, 127.6 mmol) and stirred at RT. The progress of the reaction was monitored by HPLC. After stirring for 30 min, the reaction mixture was filtered and washed with glacial acetic acid (15 mL ⋉ 3). The filtrate was poured into cold water (700 mL) and the resulting precipitate was filtered, washed with water (500 mL) and dried to obtain arjunolic acid as a white solid (3.0 g, 97%). HPLC (reverse phase) *t*_R_ = 8.3 min (Figure 1a). mp = 328 °C, [α]_D_^295^ = +60.53 (*c* 0.5 in MeOH); FTIR (neat, cm^−1^) *ν_max_* 3464 (s), 3373 (s), 2929 (s), 1706 (s), 1455 (m), 1371 (m), 1266 (m), 1193 (w). ^1^H-NMR (600 MHz, DMSO-d_6_) *δ* 12.04 (s, 1H, -COOH), 5.17 (s, 1H), 4.40 (app. s, 1H), 4.23 (d, *J* = 3.6 Hz, 1H), 4.16 (d, *J* = 3.0 Hz), 3.48 (m, 1H), 3.30 (m, 1H), 3.17 (d, *J* = 6.0 Hz, 1H), 3.03 (m, 1H), 2.74 (dd, *J_1_* = 13.2 Hz, *J_2_* = 3.6 Hz, 1H), 2.0–0.80 (terpenpoid H's, 20H), 1.12 (s, 3H), 0.91 (s, 3H), 0.87 (s, 6H), 0.70 (s, 3H), 0.53 (s, 3H). ^13^C-NMR (150 MHz, DMSO-d_6_) *δ* 178.6, 143.9, 121.5, 75.5, 67.4, 63.9, 47.1, 46.7, 46.0, 45.7, 45.5, 42.5, 41.4, 40.8, 37.4, 33.3, 32.9, 32.1, 31.9, 30.4, 27.2, 25.7, 23.4, 23.0, 22.0, 17.5, 16.9, 16.8, 13.8. HRMS(ESI) *m/z* 511.3399 [M + Na^+^, C_30_H_48_O_5_Na requires 511.3393).

**Asiatic acid **(**2**): Methyl asiatate (see Supporting Information File 1, 1.10 g, 2.21 mmol) dissolved in dry DMF (11.0 mL) was treated with LiBr (1.92 g, 22.08 mmol) and heated at 145 °C for 30 h. The volatiles were removed under reduced pressure and the crude product was purified by column chromatography to yield asiatic acid as a white solid (0.97 g, 90%). HPLC (reverse phase) *t*_R_ = 9.0 min (Figure 1c). mp = 240–242 °C. [α]_D_^298^ = +53 (*c* 0.5 in MeOH). FTIR (neat, cm^−1^) *ν_max_* 3467 (s), 3393 (s), 2938 (s), 1692 (s), 1453 (m), 1382 (m), 1267 (m), 1195 (w), 1036 (s). ^1^H-NMR (600 MHz, DMSO-d_6_) *δ* 5.12 (s, 1H), 4.50 (s, 1H), 4.34 (s, 1H), 4.21 (s, 1H), 3.29 (d, *J* = 10.2 Hz, 1H), 3.16 (d, *J* = 9.6 Hz, 1H), 3.03 (d, *J* = 10.8 Hz, 1H), 2.0–0.80 (terpenoid H's, 20H), 1.03 (s, 3H), 0.91 (s, 3H), 0.905 (d, *J* = 9.0 Hz, 3H), 0.803 (d, *J* = 6.0 Hz, 3H), 0.72 (s, 3H), 0.52 (s, 3H). ^13^C-NMR (150 MHz, DMSO-d_6_) *δ* 178.7, 138.5, 124.7, 75.7, 67.7, 64.1, 52.6, 47.2, 47.1, 46.2, 42.7, 42.0, 37.5, 36.6, 32.4, 31.5, 30.4, 29.2, 27.7, 25.6, 24.0, 23.5, 23.2, 22.3, 21.3, 17.6, 17.3, 17.1, 14.2, 14.0. HRMS(ESI) *m/z* 511.3399 [M + Na^+^, C_30_H_48_O_5_Na requires 511.3393).

### Selected Crystallographic Data of 3

Colorless prism, 0.48 ⋉ 0.22 ⋉ 0.18 mm^3^, orthorhombic, *P*2_1_2_1_2_1_, *a* = 12.4953(1), *b* = 13.6073(1), *c* = 16.2243(1) Å, *V* = 2758.57(3) Å^3^, *ρ*_calc_ = 1.367 g cm^−3^, 2*θ*_max_ = 128.28°, *λ* = 1.54178 Å, *T* = 110 K, 19822 measured reflections, 4494 independent reflections (*R*_int_ = 0.0263), 4447 observed reflections (*I*>2σ(*I*)), *μ* = 2.327 mm^−1^, semi-empirical absorption correction, *T*_min_ = 0.35, *T*_max_ = 0.66, 335 parameters, *R*1(*I*>2σ(*I*)) = 0.0246, *wR*_2_(all data) = 0.0653, Flack-parameter −0.015(10), max./min. residual electron density 0.329/−0.412 eÅ^−3^. Intensity data for **3** were collected on an Oxford Diffraction Xcalibur2 CCD employing the ω scan method using Cu*K*_α_ radiation. The data were corrected for Lorentz, polarization and absorption (multi-scan, compound **3** only) effects. **3** was solved by using direct methods (SHELXS-97) and refined by using a full-matrix least-squares refinement procedure (SHELXL-97). The protons were refined with a riding model.

## Supporting Information

File 1 Experimental data. An improved method for the isolation of the triterpenic acids, additional experimental procedures, copies of ^1^H-NMR, ^13^C-NMR, DEPT, HRMS spectra and Gel micrographs.

File 2 CIF data of arjuna-bromolactone **3**
