# Supplementary material for: A simple route for renewable nano-sized arjunolic and asiatic acids and self-assembly of arjuna-bromolactone
Source: Beilstein J Org Chem. 2008 Jul 9;4:24. doi: 10.3762/bjoc.4.24 (PMC2511022; doi:10.3762/bjoc.4.24)
Supplement: File 1 — Experimental data. An improved method for the isolation of the triterpenic acids, additional experimental procedures, copies of 1H-NMR, 13C-NMR, DEPT, HRMS spectra and Gel micrographs. [file Beilstein_J_Org_Chem-04-24-s001.pdf]

Supporting Information File 1

Title: Experimental Data

A simple route for renewable nano-sized arjunolic and asiatic acids and self-assembly of arjuna-bromolactone

Braja G. Bag[*,1], Partha P. Dey[1], Shaishab K. Dinda[1], William S. Sheldrick[*,2], Iris M. Oppel[2]

[1] Department of Chemistry and Chemical Technology, Vidyasagar University,
Midnapore 721 102, India
E-mail: bgopalbag@yahoo.co.in
[2] Lehrstuhl fuer Analytische Chemie, Ruhr University,
D 44780 Bochum, Germany
E-mail: william.sheldrick@ruhr-uni-bochum.de

Table of Contents

1. Isolation of the mixture of triterpenic acids and selected data 2, 3
2. ^{1}H-NMR and ^{13}C-NMR spectra of arjunolic acid (**1**) 4
3. DEPT and HRMS spectra of arjunolic acid (**1**) 5
4. ^{1}H-NMR and ^{13}C-NMR spectra of asiatic acid (**2**) 6
5. HRMS spectra of asiatic acid **2** and ^{1}H-NMR of arjuna-bromolactone **3** 7
6. ^{13}C-NMR and DEPT spectra of arjuna-bromolactone **3** 8
7. HRMS of arjuna-bromolactone **3** and ^{1}H-NMR spectrum of Me-asiatate (**4**) 9
8. ^{13}C-NMR and DEPT spectra of Me-asiatate (**4**) 10
9. HRMS of Me-asiatate (**4**) 11
10. Gelation tests of arjuna-bromolactone **3** 12

Mixture of Triterpenic acids (from *Terminalia Arjuna*):

The saw-dust used for the extraction of triterpenic acids was from *Terminalia arjuna* timber of approximate age 90 – 100 years (length 4 m and average diameter of 80 cm). Dried and finely powdered saw-dust from *Terminalia arjuna* (1 kg) was initially washed with petroleum ether (24 h) to remove the greasy non-polar materials. Subsequent extraction with refluxing diethyl ether (4 h x 8) using an apparatus designed in our laboratory (see supporting information of [1]) yielded a yellowish solid (40 g). This powdered solid was refluxed with acetone (700 mL) for 1 h and then concentrated to 125 mL and cooled. The precipitate was filtered (through a sintered glass funnel) to afford a whitish solid (22 g). The process was repeated once more to afford a whitish solid (18 g). The crude product was dissolved in methanol (700 mL), decolorized with charcoal and then crystallized to obtain a white crystalline solid (15 g). mp = 298 ºC. HPLC (206 nm) t_R = 8.7 min (Fig. 1b). FTIR (neat, cm^{-1}) ν_{max} 3464 (s), 3380 (s), 2925 (s), 1700 (s), 1645 (w), 1454 (m), 1373 (m), 1267 (m), 1188 (m).

Bromolactonization of the mixture of triterpenic acids 1 and 2:

A mixture of the above triterpenic acids (5.00 g, 10.23 mmol) dissolved in 90% acetic acid (100 mL) was treated with solid sodium acetate (2.5 g, 18.37 mmol). The stirred clear solution was then treated with a solution of bromine (0.63 mL, 12.27 mmol) in glacial acetic acid (65 mL) which was added dropwise over a period of 40 min at room temperature. The reaction was then stirred at room temperature for a further 1 h and then poured into an ice/water mixture (1200 mL). The white precipitate that formed was filtered and the residue was washed with distilled water (500 mL). The white solid obtained was initially dried in air and then under reduced pressure to obtain a mixture of **2** and **3** (5.4 g).

Reaction of the mixture of 2 and 3 with CH_2N_2:

A mixture of **2** and **3** (5.4 g) was suspended in methanol (10 mL) and treated with an excess of ethereal diazomethane repeatedly for complete conversion of asiatic acid (**2**) to methyl asiatate (**4**) (as revealed by HPLC analysis). The solvents were removed and the yellowish solid of the mixture of compound **3** and **4** thus obtained (5.4 g) was recrystallized from ethyl acetate. Pure arjuna-bromolactone **3** crystallized from ethyl acetate as a white crystalline solid (3.5 g) and methyl asiatate (**4**) (1.1 g) was isolated from the mother liquor. HPLC (206 nm) t_R (arjuna bromolactone) = 10.0 min, (Figure 1d), t_R (methyl asiatate) = 20.7 min (Figure 1e).

Selected data of arjuna-bromolactone 3:

MP = 230 – 233 ºC. ^{1}H-NMR (600 MHz, $CDCl_3$) δ 4.30 (d, 1H), 3.80 (br, m, 1H), 3.70 (s, 1H), 3.65 (dd, 1H, J_1 = 10.2 Hz, J_2 =3.6 Hz), 3.46 (dd, 1H, J_1 = 9.6 Hz, J_2 = 3 Hz), 3.42 (d, 1H), 2.97 (s, 1H), 2.86 (s, 1H), 2.42 - 1.00 (terpenoid protons, 18H), 1.45 (s, 3H), 1.21 (s, 3H), 1.00 (s, 3H), 0.99 (s, 3H), 0.91 (s, 3H), 0.83 (s, 3H). ^{13}C-NMR (150 MHz, $CDCl_3$) δ 178.90, 91.54, 79.36, 69.01, 68.79, 56.03, 52.28, 48.62, 45.92, 45.50, 45.42, 43.43, 42.64, 42.41, 39.88, 37.74, 34.16, 33.81, 33.26, 31.86, 30.53, 29.13, 27.46, 23.55, 21.27, 21.16, 19.04, 18.59, 17.57, 12.78. FTIR (neat, cm^{-1}) ν_{max} 3407 (s), 2938 (s), 1767 9s), 1465 (m), 1389 (m), 1359 (w), 1246 (w), 1214 (m), 1135 (m), 1107 (s), 1016 (w). $[\alpha]_D^{295}$ = + 52.63 (*c* 0.5, MeOH). HRMS (ESI): m/z calcd ($C_{30}H_{48}O_5BrNa$) 591.2467, found 591.2467 $[M + Na]^+$.

Selected data of methyl asiatate 4: mp = 222 – 224 ºC.

^{1}H-NMR (600 MHz, $CDCl_3$) δ 5.25 (s, 1H), 3.76 (m, 1H), 3.64 (m, 1H), 3.60 (s, 3H), 3.42 (m, 2H), 2.23 (d, 1H), 2.02 - 0.80 (terpenoid protons, 20H), 1.07 (s, 3H), 1.03 (s, 3H), 0.87, 0.84 (br, m, 6H), 0.74 (s, 3H). ^{13}C-NMR (150 MHz, $CDCl_3$): δ: 178.07, 138.22, 125.21, 70.59, 68.72, 52.78, 51.48, 49.10, 48.03, 42.40, 42.04, 41.99, 39.46, 39.01, 38.83, 38.12, 36.59, 32.56, 30.60, 29.69, 27.93, 24.15, 23.66, 23.22, 21.17, 18.29, 17.13, 17.03, 16.92, 16.80, 12.76. FTIR (neat, cm^{-1}) ν_{max} 3401 (s), 2946 (s), 2873 (s), 1722 (s), 1455 (s), 1389 (m), 1241 (w), 1142 (m), 1073 (s), 1048 (w). $[\alpha]_D^{298}$ = + 52 (*c* 0.046, $CHCl_3$). HRMS (ESI): m/z calcd ($C_{31}H_{50}O_5Na$) 525.3550, found 525.3556 $[M + Na]^+$.

Reference

1. Bag, B. G.; Pramanik, S. R.; Maity, G. C. *Supramol. Chem.* **2005,** *17,* 297–302. doi:10.1080/10610270500067822

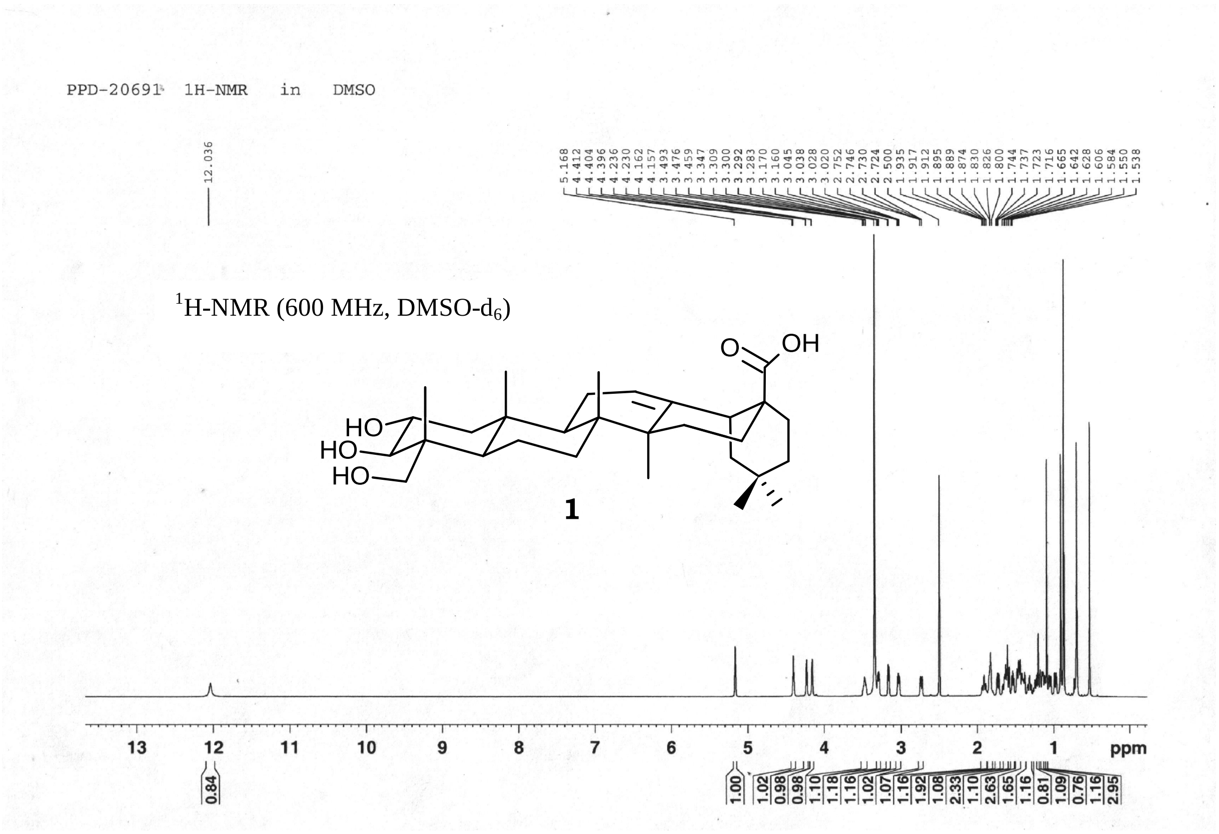
PPD-20691 1H-NMR in DMSO
12.036
5.168
4.412
4.404
4.396
4.236
4.230
4.162
4.157
3.493
3.476
3.459
3.347
3.309
3.300
3.292
3.283
3.170
3.160
3.045
3.038
3.028
3.020
2.752
2.746
2.730
2.724
2.500
1.935
1.917
1.912
1.895
1.889
1.874
1.830
1.826
1.800
1.744
1.737
1.723
1.716
1.665
1.642
1.628
1.606
1.584
1.550
1.538
^{1}H-NMR (600 MHz, DMSO-d$_6$)
O
OH
HO
HO
HO
1
13 12 11 10 9 8 7 6 5 4 3 2 1 ppm
0.84
1.00 1.02 0.98 0.98 1.10 1.18 1.16 1.02 1.07 1.16 1.92 1.08 2.33 1.10 2.63 1.65 1.16 0.81 1.09 0.76 1.16 2.95

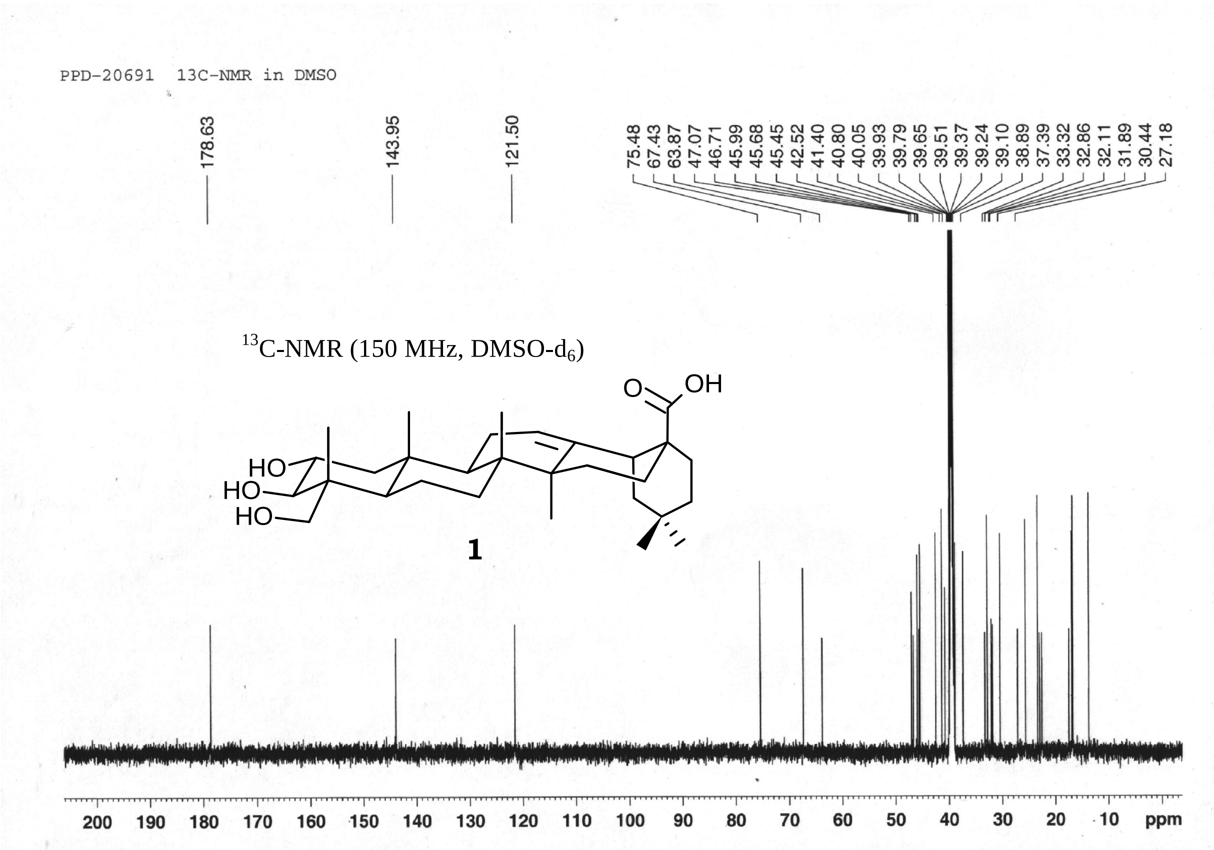
PPD-20691 13C-NMR in DMSO
178.63
143.95
121.50
75.48
67.43
63.87
47.07
46.71
45.99
45.68
45.45
42.52
41.40
40.80
40.05
39.93
39.79
39.65
39.51
39.37
39.24
39.10
38.89
37.39
33.32
32.86
32.11
31.89
30.44
27.18
^{13}C-NMR (150 MHz, DMSO-d$_6$)
O
OH
HO
HO
HO
1
200 190 180 170 160 150 140 130 120 110 100 90 80 70 60 50 40 30 20 10 ppm

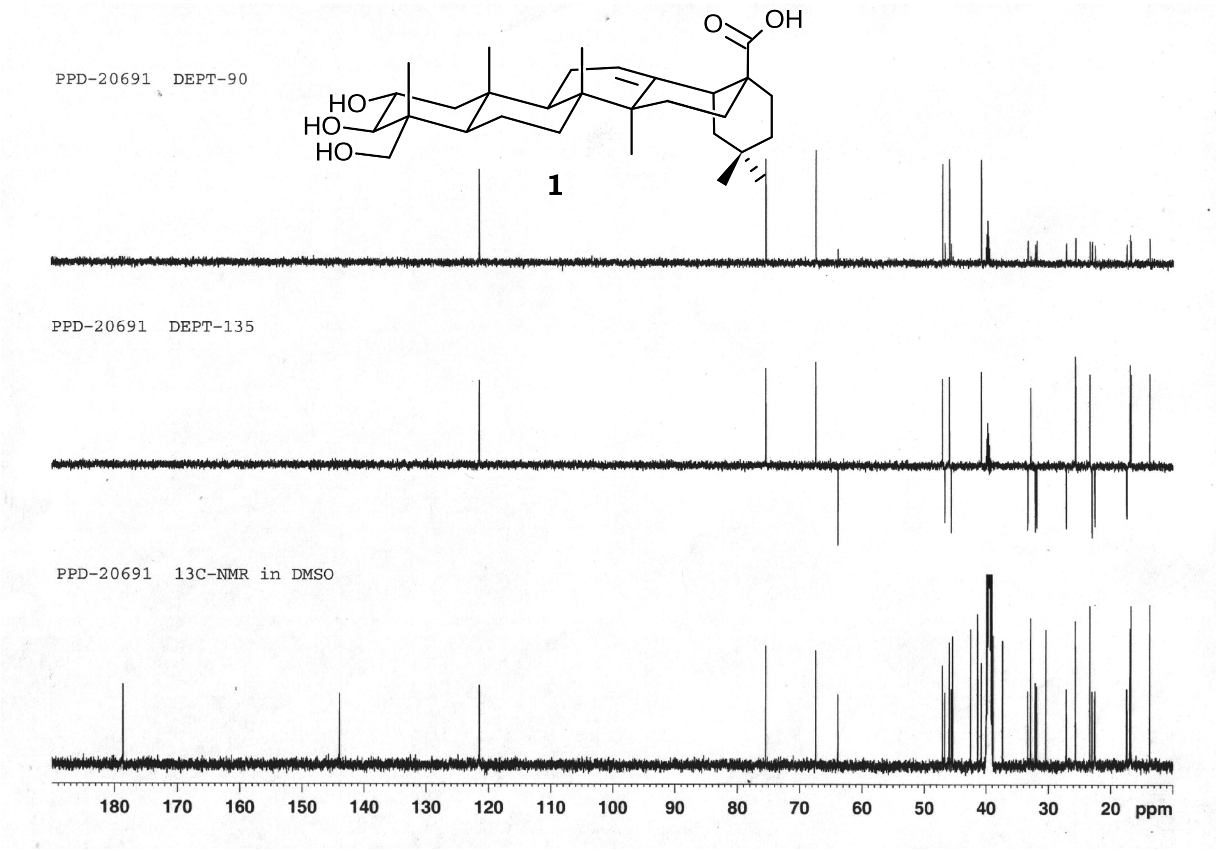
O OH
PPD-20691 DEPT-90
HO
HO
HO
1
PPD-20691 DEPT-135
PPD-20691 13C-NMR in DMSO
180 170 160 150 140 130 120 110 100 90 80 70 60 50 40 30 20 ppm

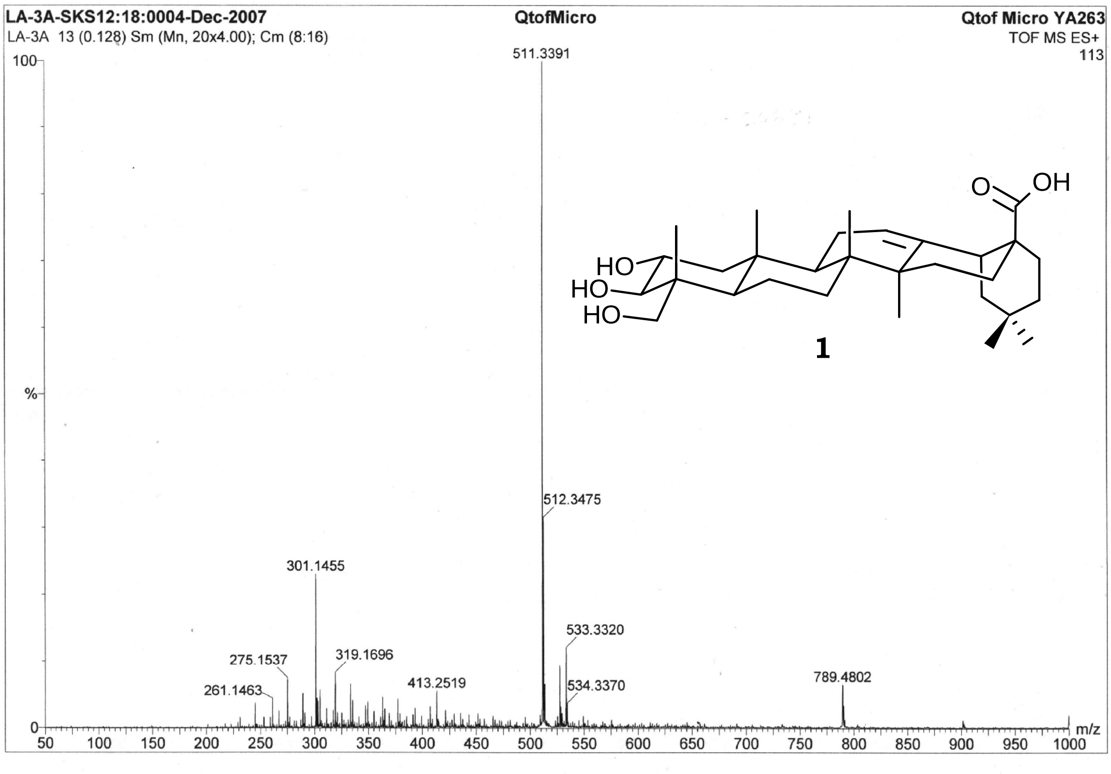
LA-3A-SKS12:18:0004-Dec-2007
QtofMicro
Qtof Micro YA263
LA-3A 13 (0.128) Sm (Mn, 20x4.00); Cm (8:16)
TOF MS ES+
113
100
%
0
511.3391
512.3475
301.1455
533.3320
275.1537
319.1696
261.1463
413.2519
534.3370
789.4802
O OH
HO
HO
HO
1
50 100 150 200 250 300 350 400 450 500 550 600 650 700 750 800 850 900 950 1000 m/z

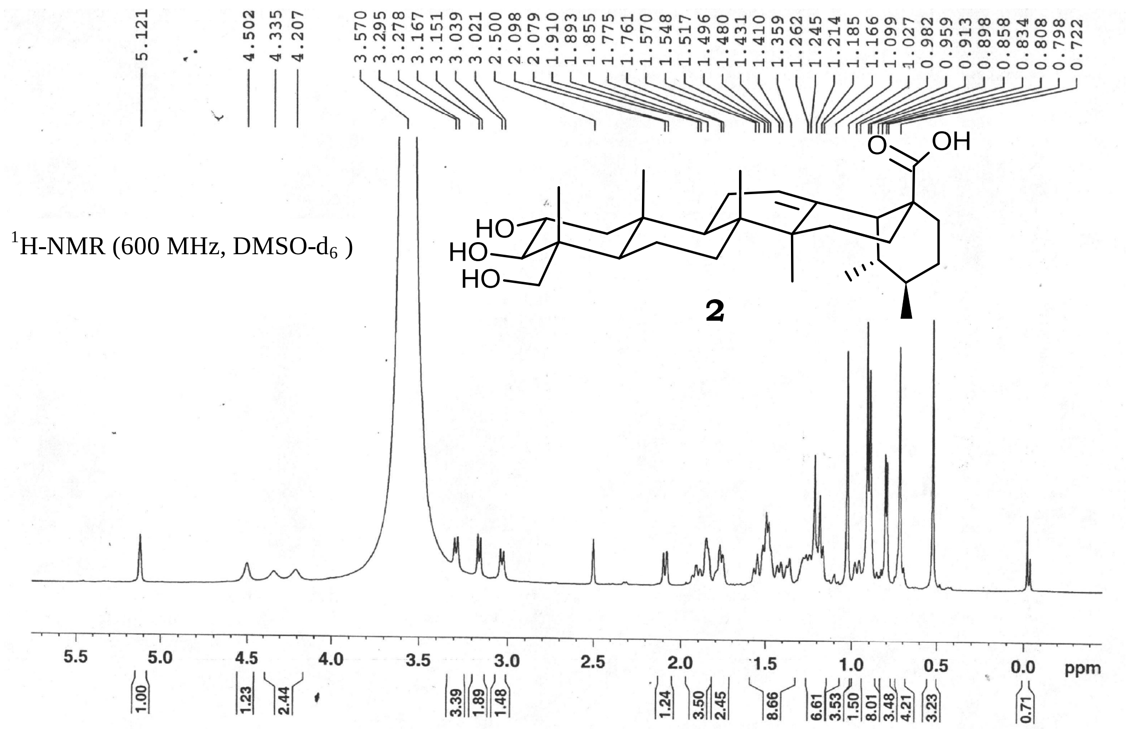
5.121
4.502
4.335
4.207
3.570
3.295
3.278
3.167
3.151
3.039
3.021
2.500
2.098
2.079
1.910
1.893
1.855
1.775
1.761
1.570
1.548
1.517
1.496
1.480
1.431
1.410
1.359
1.262
1.245
1.214
1.185
1.166
1.099
1.027
0.982
0.959
0.913
0.898
0.858
0.834
0.808
0.798
0.722
1H-NMR (600 MHz, DMSO-d6)
O
OH
HO
HO
HO
2
5.5
5.0
4.5
4.0
3.5
3.0
2.5
2.0
1.5
1.0
0.5
0.0
ppm
1.00
1.23
2.44
3.39
1.89
1.48
1.24
3.50
2.45
8.66
6.61
3.53
1.50
8.01
3.48
4.21
3.23
0.71

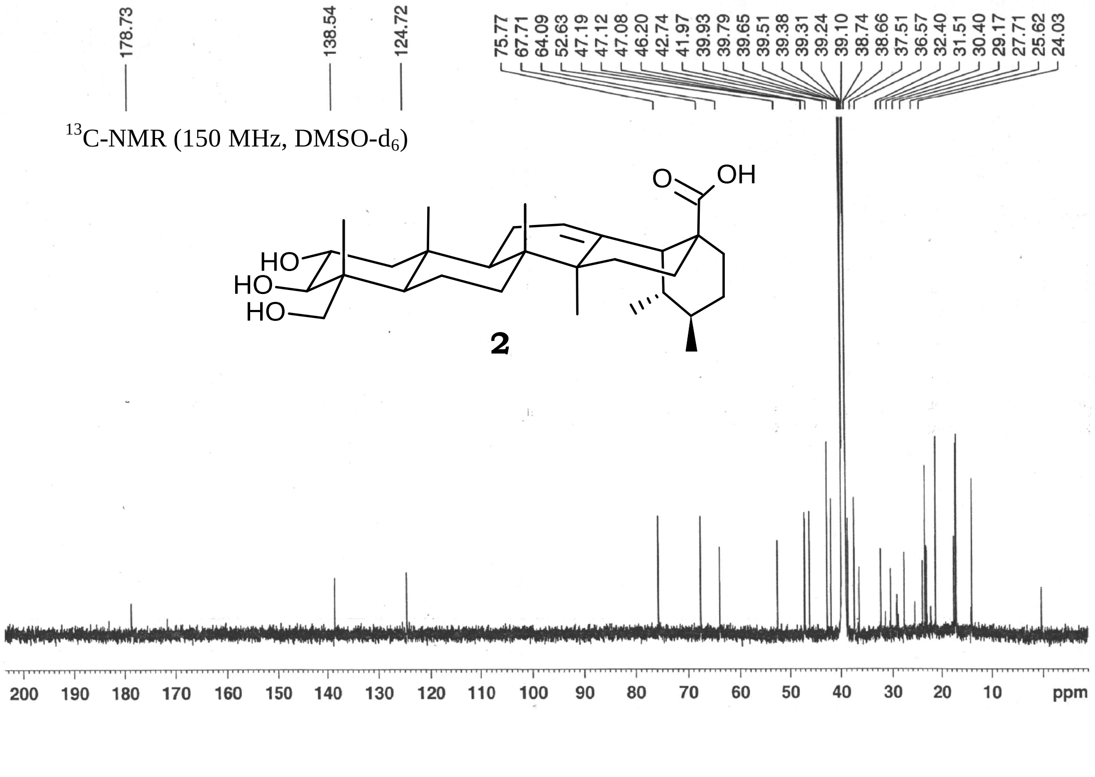
178.73
138.54
124.72
75.77
67.71
64.09
52.63
47.19
47.12
47.08
46.20
42.74
41.97
39.93
39.79
39.65
39.51
39.38
39.31
39.24
39.10
38.74
38.66
37.51
36.57
32.40
31.51
30.40
29.17
27.71
25.62
24.03
13C-NMR (150 MHz, DMSO-d6)
O
OH
HO
HO
HO
2
200
190
180
170
160
150
140
130
120
110
100
90
80
70
60
50
40
30
20
10
ppm

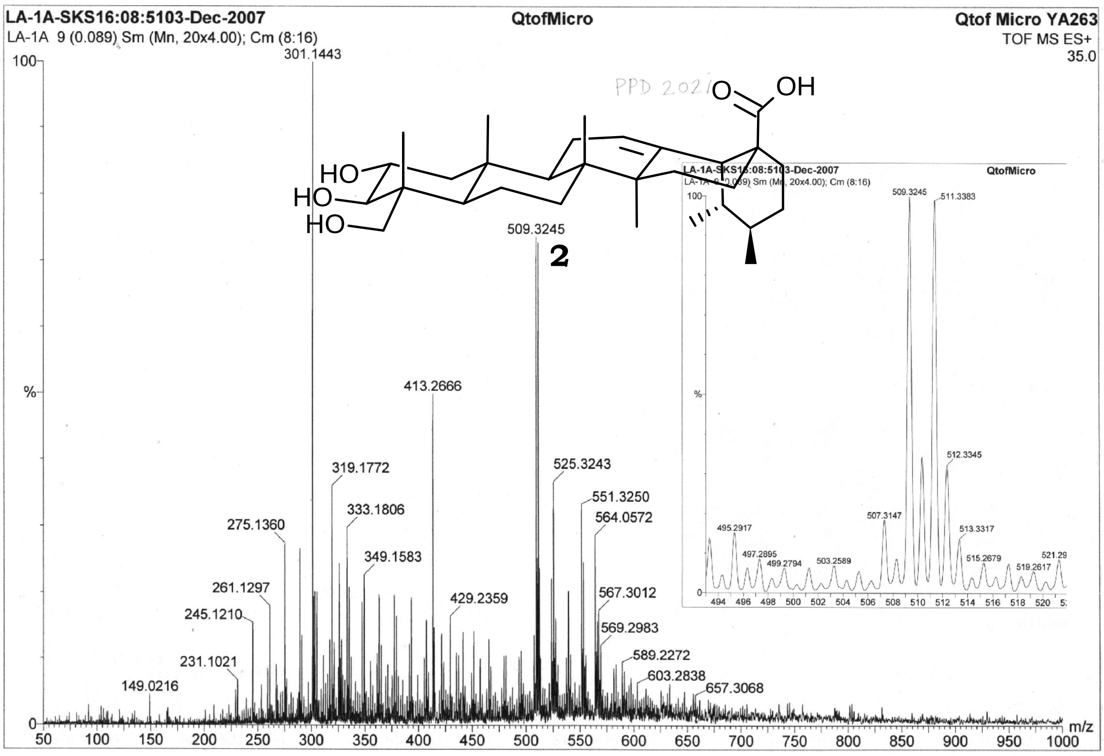

LA-1A-SKS16:08:5103-Dec-2007
QtofMicro
Qtof Micro YA263
LA-1A 9 (0.089) Sm (Mn, 20x4.00); Cm (8:16)
TOF MS ES+
35.0
301.1443
509.3245
413.2666
319.1772
525.3243
551.3250
564.0572
333.1806
275.1360
349.1583
261.1297
429.2359
567.3012
245.1210
569.2983
231.1021
589.2272
603.2838
149.0216
657.3068
%
m/z
HO
HO
HO
O
OH
2
511.3383
512.3345
507.3147
495.2917
513.3317
497.2895
499.2794
503.2589
515.2679
519.2617

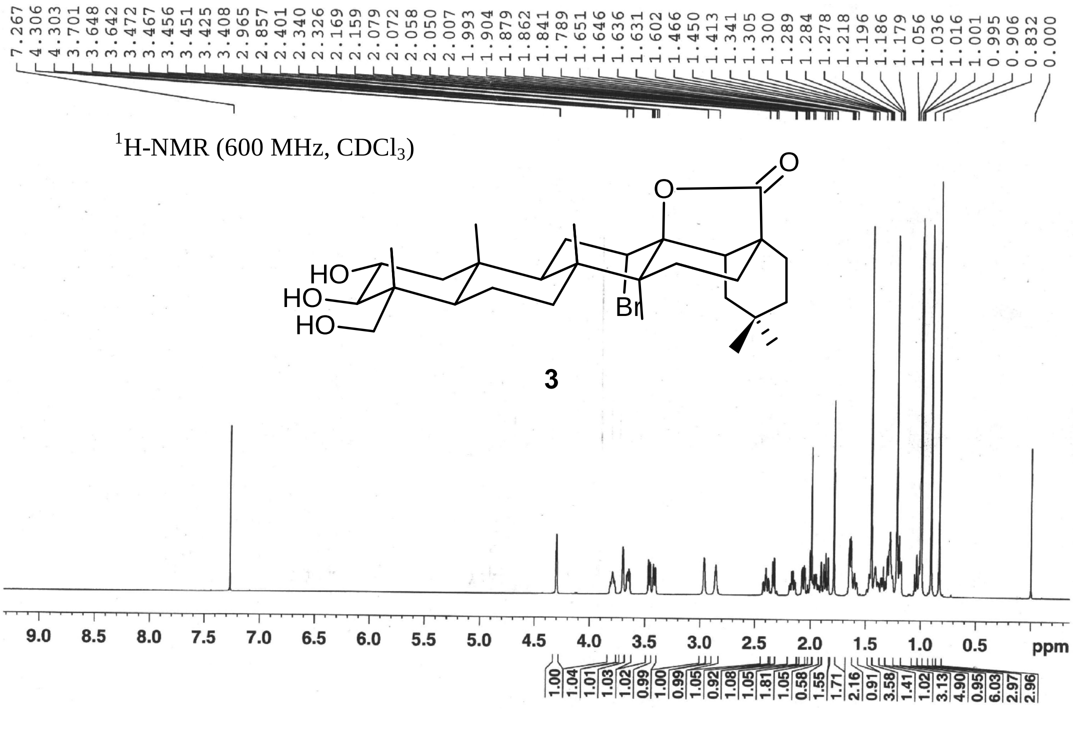

7.267
4.306
4.303
3.701
3.648
3.642
3.472
3.467
3.456
3.451
3.425
3.408
2.965
2.857
2.401
2.340
2.326
2.169
2.159
2.079
2.072
2.058
2.050
2.007
1.993
1.904
1.879
1.862
1.841
1.789
1.651
1.646
1.636
1.631
1.602
1.466
1.450
1.413
1.341
1.305
1.300
1.289
1.284
1.278
1.218
1.196
1.186
1.179
1.056
1.036
1.016
1.001
0.995
0.906
0.832
0.000
1H-NMR (600 MHz, CDCl3)
HO
HO
HO
Br
O
O
3
ppm

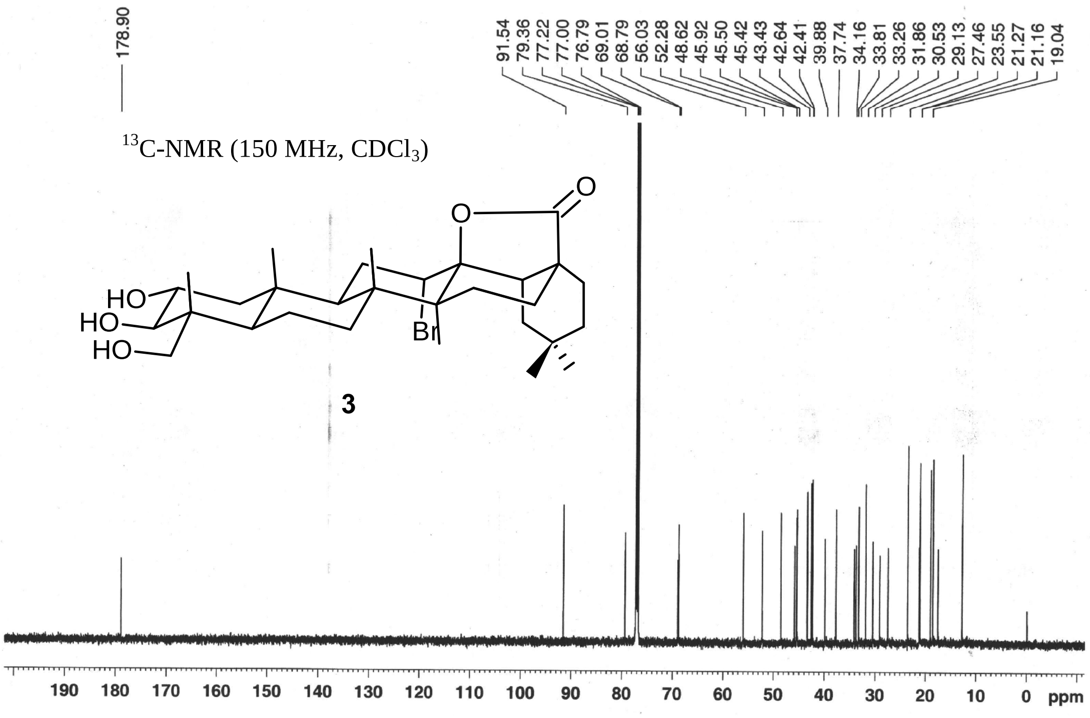

178.90
91.54 79.36 77.22 77.00 76.79 69.01 68.79 56.03 52.28 48.62 45.92 45.50 45.42 43.43 42.64 42.41 39.88 37.74 34.16 33.81 33.26 31.86 30.53 29.13 27.46 23.55 21.27 21.16 19.04
13C-NMR (150 MHz, CDCl3)
O
O
HO
HO
HO
Br
3
190 180 170 160 150 140 130 120 110 100 90 80 70 60 50 40 30 20 10 0 ppm

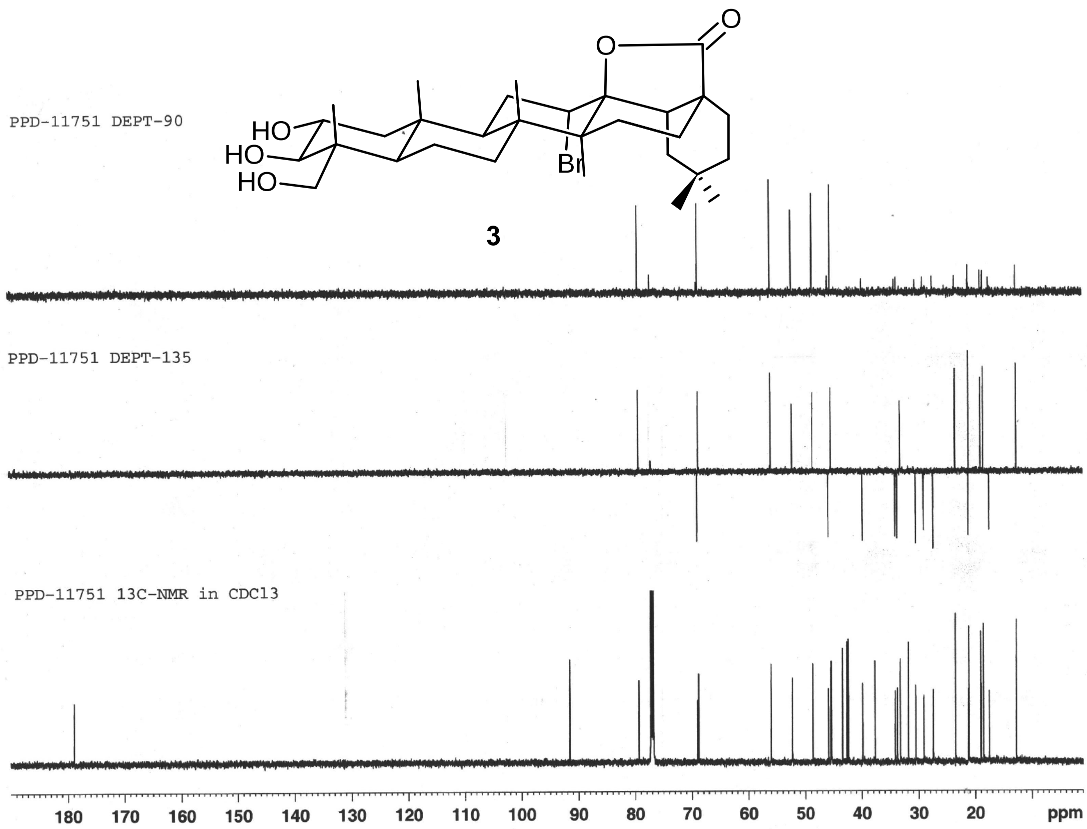

PPD-11751 DEPT-90
O
O
HO
HO
HO
Br
3
PPD-11751 DEPT-135
PPD-11751 13C-NMR in CDCl3
180 170 160 150 140 130 120 110 100 90 80 70 60 50 40 30 20 ppm

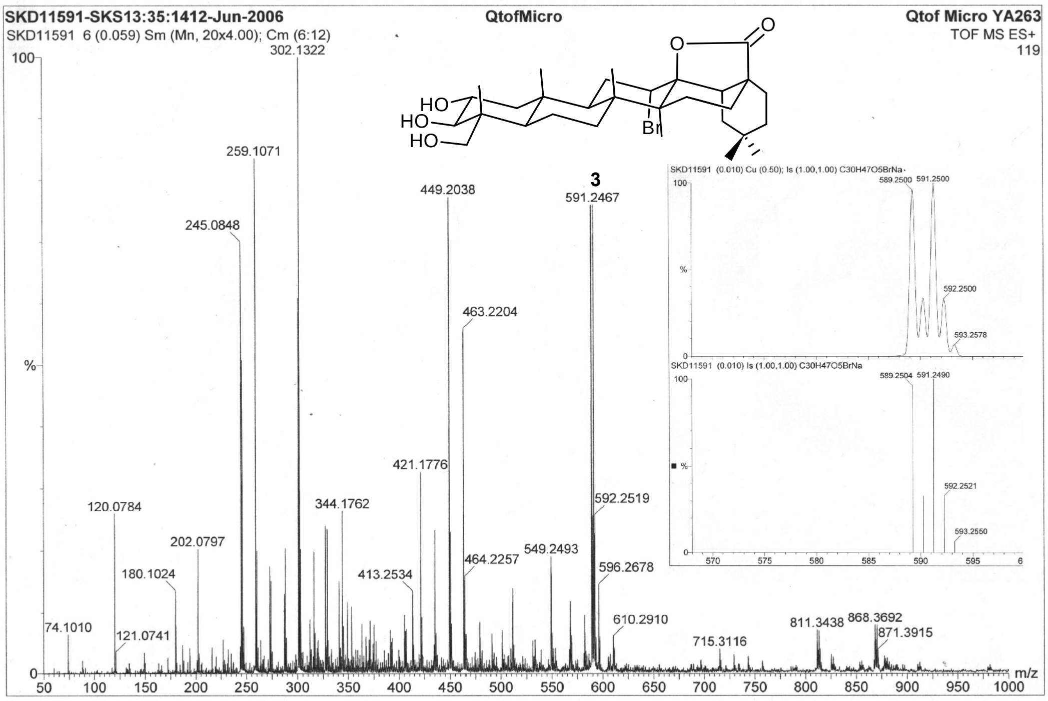
SKD11591-SKS13:35:1412-Jun-2006
SKD11591 6 (0.059) Sm (Mn, 20x4.00); Cm (6:12)
QtofMicro
Qtof Micro YA263
TOF MS ES+
119
3
302.1322
259.1071
245.0848
449.2038
591.2467
463.2204
421.1776
344.1762
120.0784
202.0797
180.1024
413.2534
464.2257
549.2493
592.2519
596.2678
610.2910
74.1010
121.0741
715.3116
811.3438
868.3692
871.3915
m/z
SKD11591 (0.010) Cu (0.50); Is (1.00,1.00) C30H47O5BrNa
SKD11591 (0.010) Is (1.00,1.00) C30H47O5BrNa

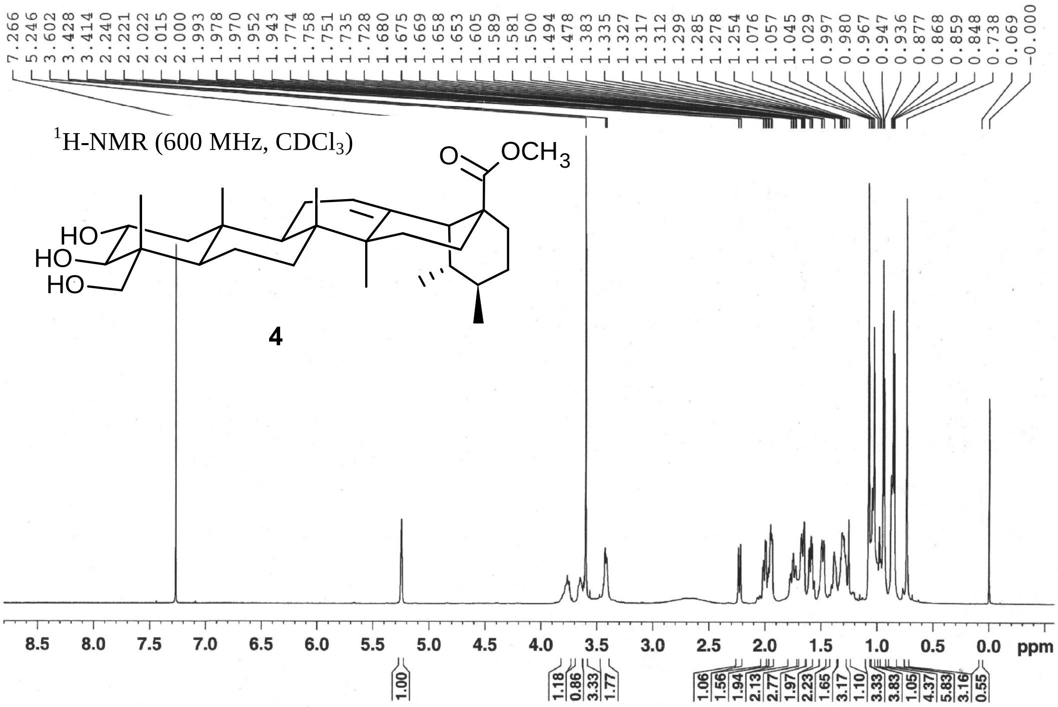
1H-NMR (600 MHz, CDCl3)
4
ppm

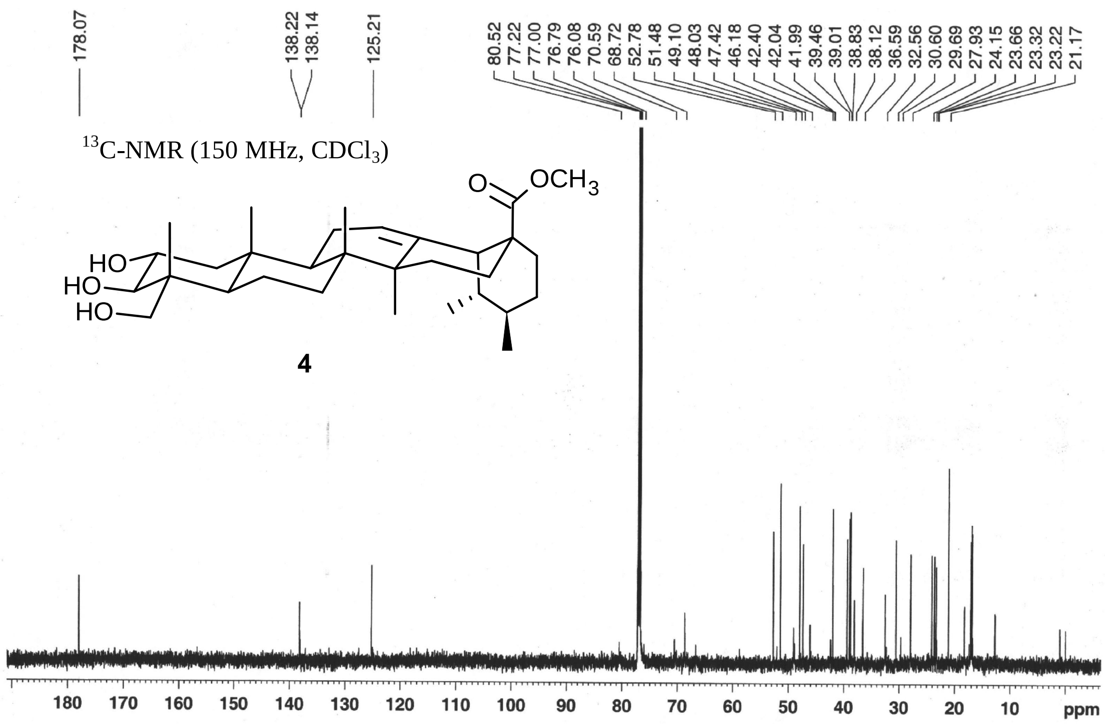
178.07
138.22
138.14
125.21
80.52
77.22
77.00
76.79
76.08
70.59
68.72
52.78
51.48
49.10
48.03
47.42
46.18
42.40
42.04
41.99
39.46
39.01
38.83
38.12
36.59
32.56
30.60
29.69
27.93
24.15
23.66
23.32
23.22
21.17
^{13}C-NMR (150 MHz, $CDCl_3$)
O
OCH_3
HO
HO
HO
4
180 170 160 150 140 130 120 110 100 90 80 70 60 50 40 30 20 10 ppm

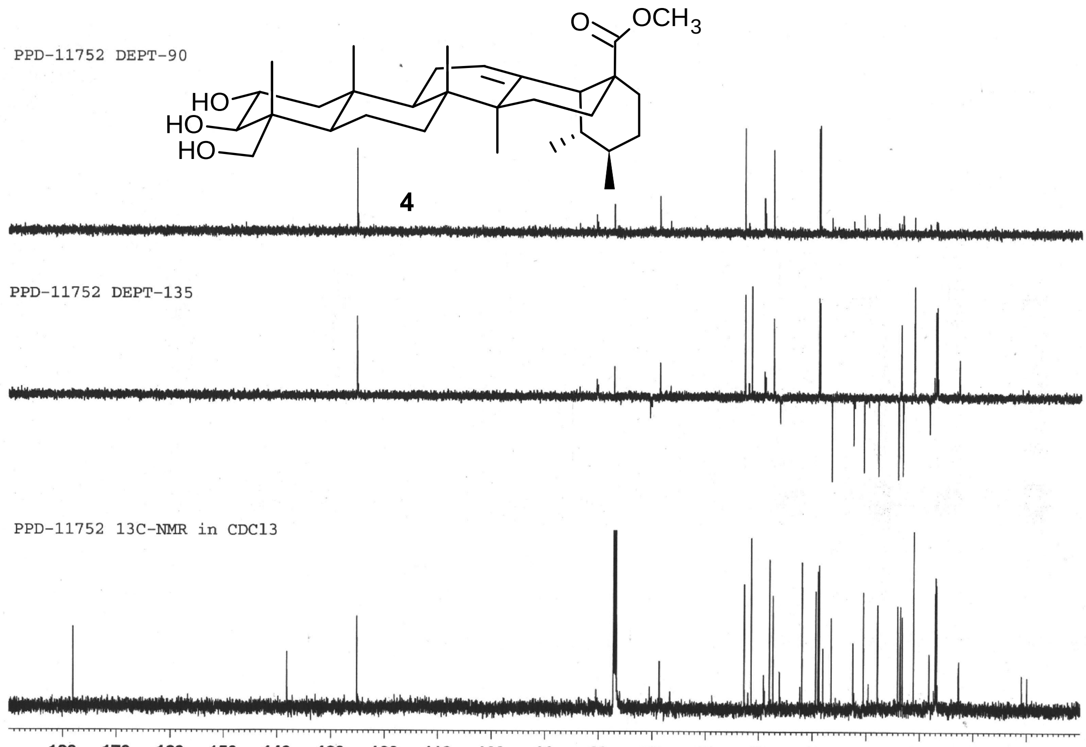
PPD-11752 DEPT-90
O
OCH_3
HO
HO
HO
4
PPD-11752 DEPT-135
PPD-11752 13C-NMR in CDCl3
180 170 160 150 140 130 120 110 100 90 80 70 60 50 40 30 20 10 0 ppm

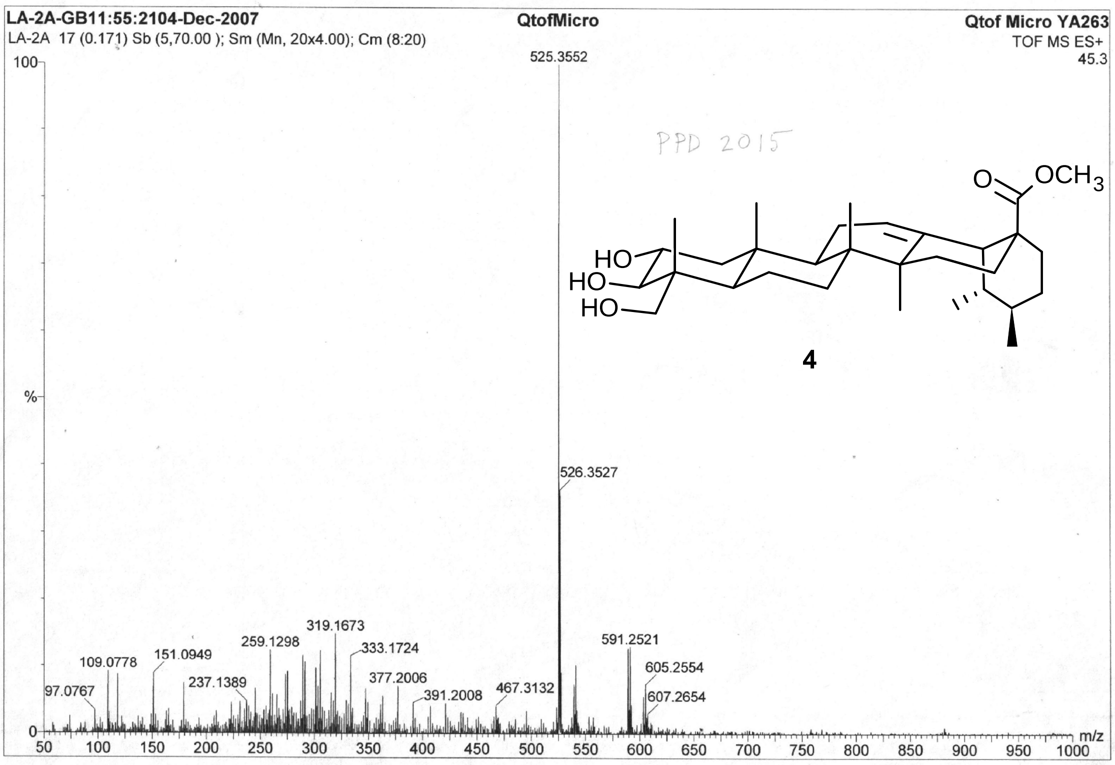
LA-2A-GB11:55:2104-Dec-2007
QtofMicro
Qtof Micro YA263
LA-2A 17 (0.171) Sb (5,70.00); Sm (Mn, 20x4.00); Cm (8:20)
TOF MS ES+
45.3
100
%
0
525.3552
526.3527
319.1673
259.1298
333.1724
109.0778
151.0949
237.1389
377.2006
97.0767
391.2008
467.3132
591.2521
605.2554
607.2654
m/z
50
100
150
200
250
300
350
400
450
500
550
600
650
700
750
800
850
900
950
1000
PPD 2015
O
OCH3
HO
HO
HO

4

Gelation Tests: The arjuna-bromolactone **3** (ca. 5 mg) was dissolved by heating with an organic solvent in a vial. The clear solution thus obtained was allowed to cool to RT and examined visually. When the vial could be turned upside down without significant flow of the medium, we called it a gel. Minimum gel concentration (mgc) was measured by repeating the gelation experiments at different initial concentrations and allowing the solutions to cool to room temperature. The minimum concentration needed for gelation at room temperature was termed as the minimum gel concentration (mgc). The gel to sol transition temperature (T_{gel}) was measured by the ball drop method.

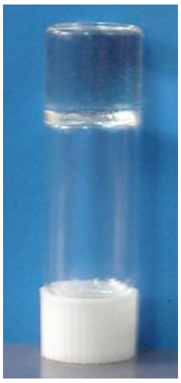

Figure 1: Inverted vial containing a gel of compound **3** in mesitylene (0.65% w/v). Photograph was taken after 4 h of cooling at RT).

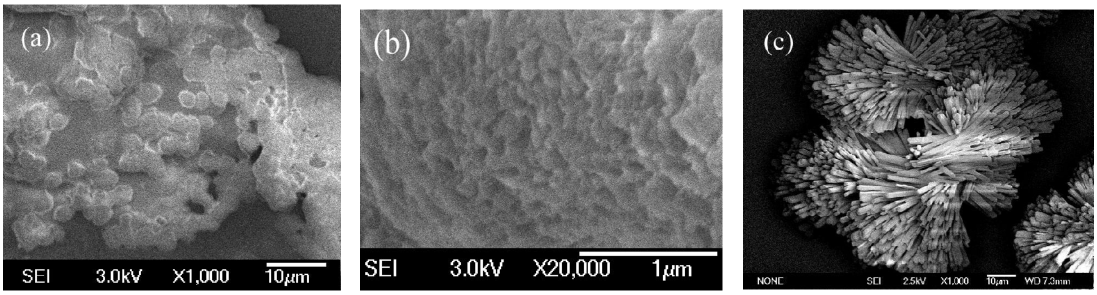

Figure 2: Scanning electron micrographs of the dried gels in mesitylene (2.25% w/v, (a) and (b)) and *o*-xylene (1.6% w/v, (c)) after sputter coating with Pt.
