# Supplementary material for: A simple route for renewable nano-sized arjunolic and asiatic acids and self-assembly of arjuna-bromolactone
Source: Beilstein J Org Chem. 2008 Jul 9;4:24. doi: 10.3762/bjoc.4.24 (PMC2511022; doi:10.3762/bjoc.4.24)
Supplement: File 2 — CIF data of arjuna-bromolactone 3 [file Beilstein_J_Org_Chem-04-24-s002.pdf]

Supporting Information File 2

Title: CIF data of arjuna-bromolactone 3

A simple route for renewable nano-sized arjunolic and asiatic acids and self-assembly of arjuna-bromolactone

Braja G. Bag[*,1], Partha P. Dey[1], Shaishab K. Dinda[1], William S. Sheldrick[*,2], Iris M. Oppel[2]

[1] Department of Chemistry and Chemical Technology, Vidyasagar University,
Midnapore 721 102, India
E-mail: bgopalbag@yahoo.co.in
[2] Lehrstuhl fuer Analytische Chemie, Ruhr University,
D 44780 Bochum, Germany
E-mail: william.sheldrick@ruhr-uni-bochum.de

```
data_p212121

_audit_creation_method SHELXL-97
_chemical_name_systematic
;
?
;
_chemical_name_common ?
_chemical_melting_point ?
_chemical_formula_moiety ?
_chemical_formula_sum
'C30 H47 Br O5'
_chemical_formula_weight 567.59

loop_
_atom_type_symbol
_atom_type_description
_atom_type_scat_dispersion_real
_atom_type_scat_dispersion_imag
_atom_type_scat_source
'C' 'C' 0.0181 0.0091
'International Tables Vol C Tables 4.2.6.8 and 6.1.1.4'
'H' 'H' 0.0000 0.0000
'International Tables Vol C Tables 4.2.6.8 and 6.1.1.4'
'O' 'O' 0.0492 0.0322
'International Tables Vol C Tables 4.2.6.8 and 6.1.1.4'
'Br' 'Br' -0.6763 1.2805
'International Tables Vol C Tables 4.2.6.8 and 6.1.1.4'

_symmetry_cell_setting orthorhombic
_symmetry_space_group_name_H-M P2(1)2(1)2(1)

loop_
_symmetry_equiv_pos_as_xyz
'x, y, z'
'-x+1/2, -y, z+1/2'
'-x, y+1/2, -z+1/2'
'x+1/2, -y+1/2, -z'

_cell_length_a 12.49530(10)
_cell_length_b 13.60730(10)
_cell_length_c 16.22430(10)
_cell_angle_alpha 90.00
```

_cell_angle_beta 90.00
_cell_angle_gamma 90.00
_cell_volume 2758.57(3)
_cell_formula_units_Z 4
_cell_measurement_temperature 110(2)
_cell_measurement_reflns_used 15642
_cell_measurement_theta_min 2.7
_cell_measurement_theta_max 64.0

_exptl_crystal_description prism
_exptl_crystal_colour colourless
_exptl_crystal_size_max 0.48
_exptl_crystal_size_mid 0.22
_exptl_crystal_size_min 0.18
_exptl_crystal_density_meas ?
_exptl_crystal_density_diffrn 1.367
_exptl_crystal_density_method 'not measured'
_exptl_crystal_F_000 1208
_exptl_absorpt_coefficient_mu 2.327
_exptl_absorpt_correction_type 'multi-scan'
_exptl_absorpt_correction_T_min 0.35
_exptl_absorpt_correction_T_max 0.66
_exptl_absorpt_process_details
;
CrysAlis RED, Oxford Diffraction Ltd.,
Version 1.171.32.4 (release 27-04-2006 CrysAlis171 .NET)
(compiled Apr 27 2007,17:53:11)
Empirical absorption correction using spherical harmonics,
implemented in SCALE3 ABSPACK scaling algorithm.
;

_diffrn_ambient_temperature 110(2)
_diffrn_radiation_wavelength 1.54178
_diffrn_radiation_type CuK\a
_diffrn_radiation_source 'Enhance (Cu) X-ray Source'
_diffrn_radiation_monochromator graphite
_diffrn_measurement_device_type '1900 frames in \w and 15 sec '
_diffrn_measurement_method ?
_diffrn_detector_area_resol_mean ?
_diffrn_standards_number ?
_diffrn_standards_interval_count ?
_diffrn_standards_interval_time ?
_diffrn_standards_decay_% ?
_diffrn_reflns_number 19822
_diffrn_reflns_av_R_equivalents 0.0263

_diffrn_reflns_av_sigmaI/netI 0.0170
_diffrn_reflns_limit_h_min -14
_diffrn_reflns_limit_h_max 10
_diffrn_reflns_limit_k_min -15
_diffrn_reflns_limit_k_max 15
_diffrn_reflns_limit_l_min -18
_diffrn_reflns_limit_l_max 18
_diffrn_reflns_theta_min 8.27
_diffrn_reflns_theta_max 64.14
_reflns_number_total 4494
_reflns_number_gt 4447
_reflns_threshold_ex-pression >2sigma(I)

_computing_data_collection 'CrysAlis CCD Oxford diffraction'
_computing_cell_refinement 'CrysAlis RED Oxford diffraction'
_computing_data_reduction 'CrysAlis RED Oxford diffraction'
_computing_structure_solution 'SHELXS-97 (Sheldrick, 1990)'
_computing_structure_refinement 'SHELXL-97 (Sheldrick, 1997)'
_computing_molecular_graphics 'SHELXL-97'
_computing_publication_material 'SHELXL-97'

_refine_special_details
;
Refinement of F^2^ against ALL reflections. The weighted R-factor wR and goodness of fit S are based on F^2^, conventional R-factors R are based on F, with F set to zero for negative F^2^. The threshold ex-pression of F^2^ > 2sigma(F^2^) is used only for calculating R-factors(gt) etc. and is not relevant to the choice of reflections for refinement. R-factors based on F^2^ are statistically about twice as large as those based on F, and R-factors based on ALL data will be even larger.
;

_refine_ls_structure_factor_coef Fsqd
_refine_ls_matrix_type full
_refine_ls_weighting_scheme calc
_refine_ls_weighting_details
'calc w=1/[\s^2^(Fo^2^)+(0.0450P)^2^+0.0344P] where P=(Fo^2^+2Fc^2^)/3'
_atom_sites_solution_primary direct
_atom_sites_solution_secondary difmap
_atom_sites_solution_hydrogens geom
_refine_ls_hydrogen_treatment constr
_refine_ls_extinction_method SHELXL
_refine_ls_extinction_coef 0.0029(2)
_refine_ls_extinction_ex-pression
'Fc^*^=kFc[1+0.001xFc^2^\l^3^/sin(2\q)]^-1/4^'
_refine_ls_abs_structure_details

'Flack H D (1983), Acta Cryst. A39, 876-881'
_refine_ls_abs_structure_Flack -0.015(10)
_refine_ls_number_reflns 4494
_refine_ls_number_parameters 335
_refine_ls_number_restraints 0
_refine_ls_R_factor_all 0.0252
_refine_ls_R_factor_gt 0.0246
_refine_ls_wR_factor_ref 0.0653
_refine_ls_wR_factor_gt 0.0648
_refine_ls_goodness_of_fit_ref 1.222
_refine_ls_restrained_S_all 1.222
_refine_ls_shift/su_max 0.003
_refine_ls_shift/su_mean 0.000

loop_
_atom_site_label
_atom_site_type_symbol
_atom_site_fract_x
_atom_site_fract_y
_atom_site_fract_z
_atom_site_U_iso_or_equiv
_atom_site_adp_type
_atom_site_occupancy
_atom_site_symmetry_multiplicity
_atom_site_calc_flag
_atom_site_refinement_flags
_atom_site_disorder_assembly
_atom_site_disorder_group
Br15 Br 0.710389(16) 0.840870(16) 0.944524(12) 0.02687(9) Uani 1 1 d . . .
O2 O 0.81337(11) 0.85665(11) 0.58520(8) 0.0218(3) Uani 1 1 d . . .
H2 H 0.8231 0.8381 0.5364 0.033 Uiso 1 1 calc R . .
O3 O 0.65962(12) 0.84278(10) 0.46046(7) 0.0206(3) Uani 1 1 d . . .
H3 H 0.6525 0.7855 0.4419 0.031 Uiso 1 1 calc R . .
O14 O 0.47785(10) 1.05203(9) 0.98869(7) 0.0124(3) Uani 1 1 d . . .
O23 O 0.41584(11) 0.72923(9) 0.55703(8) 0.0184(3) Uani 1 1 d . . .
H23 H 0.4616 0.6881 0.5412 0.028 Uiso 1 1 calc R . .
O24 O 0.37747(11) 1.13585(9) 1.07769(8) 0.0190(3) Uani 1 1 d . . .
C01 C 0.68594(14) 0.91043(14) 0.68324(11) 0.0133(4) Uani 1 1 d . . .
H01A H 0.7413 0.9513 0.7102 0.016 Uiso 1 1 calc R . .
H01B H 0.6891 0.8440 0.7081 0.016 Uiso 1 1 calc R . .
C02 C 0.71142(16) 0.90260(13) 0.59193(11) 0.0156(4) Uani 1 1 d . . .
H02 H 0.7161 0.9703 0.5682 0.019 Uiso 1 1 calc R . .
C03 C 0.62649(15) 0.84509(14) 0.54516(10) 0.0147(4) Uani 1 1 d . . .
H03 H 0.6255 0.7762 0.5666 0.018 Uiso 1 1 calc R . .
C04 C 0.51388(15) 0.88932(13) 0.55551(11) 0.0142(4) Uani 1 1 d . . .
C05 C 0.49258(15) 0.89520(13) 0.65000(11) 0.0120(4) Uani 1 1 d . . .

H05 H 0.5025 0.8262 0.6697 0.014 Uiso 1 1 calc R . .
C06 C 0.37737(15) 0.92038(15) 0.67417(11) 0.0144(4) Uani 1 1 d . . .
H06A H 0.3644 0.9914 0.6657 0.017 Uiso 1 1 calc R . .
H06B H 0.3268 0.8833 0.6389 0.017 Uiso 1 1 calc R . .
C07 C 0.35871(15) 0.89390(14) 0.76462(11) 0.0144(4) Uani 1 1 d . . .
H07A H 0.3705 0.8225 0.7720 0.017 Uiso 1 1 calc R . .
H07B H 0.2833 0.9082 0.7790 0.017 Uiso 1 1 calc R . .
C08 C 0.43284(14) 0.95059(13) 0.82415(10) 0.0110(4) Uani 1 1 d . . .
C09 C 0.42507(15) 0.90296(13) 0.91490(11) 0.0109(4) Uani 1 1 d . . .
C10 C 0.31434(14) 0.92384(14) 0.95441(11) 0.0134(4) Uani 1 1 d . . .
H10A H 0.2943 0.9929 0.9428 0.016 Uiso 1 1 calc R . .
H10B H 0.2603 0.8810 0.9279 0.016 Uiso 1 1 calc R . .
C11 C 0.31058(14) 0.90701(14) 1.04836(11) 0.0148(4) Uani 1 1 d . . .
H11A H 0.3131 0.8355 1.0596 0.018 Uiso 1 1 calc R . .
H11B H 0.2419 0.9325 1.0701 0.018 Uiso 1 1 calc R . .
C12 C 0.40396(15) 0.95772(14) 1.09413(10) 0.0131(4) Uani 1 1 d . . .
C13 C 0.51000(15) 0.91186(13) 1.06600(11) 0.0125(4) Uani 1 1 d . . .
H13 H 0.5673 0.9504 1.0944 0.015 Uiso 1 1 calc R . .
C14 C 0.51169(15) 0.94716(13) 0.97396(11) 0.0109(4) Uani 1 1 d . . .
C15 C 0.62317(14) 0.96205(13) 0.93855(11) 0.0146(4) Uani 1 1 d . . .
H15 H 0.6596 1.0126 0.9734 0.018 Uiso 1 1 calc R . .
C16 C 0.62494(15) 0.99937(14) 0.85062(10) 0.0138(4) Uani 1 1 d . . .
H16A H 0.6990 0.9960 0.8291 0.017 Uiso 1 1 calc R . .
H16B H 0.6022 1.0691 0.8498 0.017 Uiso 1 1 calc R . .
C17 C 0.55088(14) 0.93925(13) 0.79460(10) 0.0109(4) Uani 1 1 d . . .
H17 H 0.5697 0.8690 0.8056 0.013 Uiso 1 1 calc R . .
C18 C 0.57498(14) 0.95554(13) 0.70065(10) 0.0112(4) Uani 1 1 d . . .
C19 C 0.50210(17) 0.98615(14) 0.50677(11) 0.0187(4) Uani 1 1 d . . .
H19A H 0.5649 1.0277 0.5165 0.028 Uiso 1 1 calc R . .
H19B H 0.4376 1.0208 0.5251 0.028 Uiso 1 1 calc R . .
H19C H 0.4963 0.9715 0.4478 0.028 Uiso 1 1 calc R . .
C20 C 0.39463(16) 1.05879(14) 0.82140(11) 0.0152(4) Uani 1 1 d . . .
H20A H 0.3975 1.0828 0.7645 0.023 Uiso 1 1 calc R . .
H20B H 0.4413 1.0992 0.8562 0.023 Uiso 1 1 calc R . .
H20C H 0.3209 1.0628 0.8418 0.023 Uiso 1 1 calc R . .
C21 C 0.43928(17) 0.79047(14) 0.90977(12) 0.0175(4) Uani 1 1 d . . .
H21A H 0.4633 0.7654 0.9633 0.026 Uiso 1 1 calc R . .
H21B H 0.4928 0.7747 0.8676 0.026 Uiso 1 1 calc R . .
H21C H 0.3708 0.7599 0.8952 0.026 Uiso 1 1 calc R . .
C22 C 0.57790(16) 1.06626(14) 0.67793(11) 0.0166(4) Uani 1 1 d . . .
H22A H 0.6266 1.0763 0.6314 0.025 Uiso 1 1 calc R . .
H22B H 0.6030 1.1043 0.7254 0.025 Uiso 1 1 calc R . .
H22C H 0.5059 1.0881 0.6626 0.025 Uiso 1 1 calc R . .
C23 C 0.43209(16) 0.81984(14) 0.51482(11) 0.0177(4) Uani 1 1 d . . .
H23A H 0.3626 0.8544 0.5108 0.021 Uiso 1 1 calc R . .
H23B H 0.4563 0.8053 0.4580 0.021 Uiso 1 1 calc R . .

C24 C 0.41520(14) 1.05854(14) 1.05725(11) 0.0134(4) Uani 1 1 d . . .
C25 C 0.39289(16) 0.95650(15) 1.18781(11) 0.0174(4) Uani 1 1 d . . .
H25A H 0.3210 0.9808 1.2035 0.021 Uiso 1 1 calc R . .
H25B H 0.4469 1.0009 1.2125 0.021 Uiso 1 1 calc R . .
C26 C 0.40851(17) 0.85254(16) 1.22133(11) 0.0212(4) Uani 1 1 d . . .
H26A H 0.3478 0.8112 1.2031 0.025 Uiso 1 1 calc R . .
H26B H 0.4070 0.8550 1.2823 0.025 Uiso 1 1 calc R . .
C27 C 0.51364(18) 0.80363(15) 1.19378(12) 0.0223(4) Uani 1 1 d . . .
C28 C 0.52126(17) 0.80637(14) 1.09821(12) 0.0181(4) Uani 1 1 d . . .
H28A H 0.5911 0.7791 1.0806 0.022 Uiso 1 1 calc R . .
H28B H 0.4640 0.7649 1.0743 0.022 Uiso 1 1 calc R . .
C29 C 0.5121(2) 0.69546(17) 1.22029(14) 0.0324(5) Uani 1 1 d . . .
H29A H 0.5057 0.6915 1.2804 0.049 Uiso 1 1 calc R . .
H29B H 0.5786 0.6635 1.2028 0.049 Uiso 1 1 calc R . .
H29C H 0.4510 0.6623 1.1946 0.049 Uiso 1 1 calc R . .
C30 C 0.61047(18) 0.85331(18) 1.23396(13) 0.0294(5) Uani 1 1 d . . .
H30A H 0.6104 0.9235 1.2204 0.044 Uiso 1 1 calc R . .
H30B H 0.6764 0.8231 1.2133 0.044 Uiso 1 1 calc R . .
H30C H 0.6066 0.8451 1.2939 0.044 Uiso 1 1 calc R . .

loop_
_atom_site_aniso_label
_atom_site_aniso_U_11
_atom_site_aniso_U_22
_atom_site_aniso_U_33
_atom_site_aniso_U_23
_atom_site_aniso_U_13
_atom_site_aniso_U_12
Br15 0.02384(13) 0.03389(14) 0.02290(12) 0.00452(9) 0.00319(9) 0.01832(10)
O2 0.0178(7) 0.0294(8) 0.0180(6) -0.0045(6) 0.0071(5) 0.0037(6)
O3 0.0317(8) 0.0208(7) 0.0093(6) -0.0035(6) 0.0072(5) 0.0024(6)
O14 0.0153(6) 0.0102(6) 0.0116(6) -0.0017(5) 0.0020(5) 0.0004(5)
O23 0.0196(7) 0.0173(7) 0.0184(6) -0.0028(6) -0.0007(6) -0.0010(5)
O24 0.0265(7) 0.0136(7) 0.0169(6) -0.0025(5) 0.0033(6) 0.0045(6)
C01 0.0126(9) 0.0158(9) 0.0114(8) -0.0019(7) 0.0020(7) -0.0011(7)
C02 0.0170(9) 0.0162(9) 0.0135(8) -0.0001(7) 0.0049(8) 0.0002(8)
C03 0.0223(9) 0.0147(9) 0.0071(8) -0.0010(8) 0.0035(7) 0.0016(8)
C04 0.0202(9) 0.0149(9) 0.0076(8) -0.0003(7) 0.0016(8) 0.0004(7)
C05 0.0146(9) 0.0124(8) 0.0090(9) -0.0013(7) -0.0002(7) 0.0008(7)
C06 0.0148(9) 0.0186(9) 0.0097(8) -0.0008(7) -0.0025(7) -0.0005(8)
C07 0.0096(8) 0.0224(10) 0.0112(8) -0.0001(7) -0.0008(7) -0.0020(8)
C08 0.0122(9) 0.0139(9) 0.0068(8) 0.0002(7) -0.0001(7) 0.0003(7)
C09 0.0128(9) 0.0108(9) 0.0093(8) -0.0003(7) -0.0006(7) -0.0011(7)
C10 0.0128(9) 0.0175(9) 0.0098(8) -0.0019(7) 0.0002(7) -0.0004(7)
C11 0.0143(9) 0.0183(9) 0.0118(8) -0.0018(7) 0.0015(7) -0.0007(7)
C12 0.0142(9) 0.0163(9) 0.0089(8) -0.0004(7) 0.0006(7) 0.0010(8)

C13 0.0134(8) 0.0149(9) 0.0094(8) 0.0002(7) -0.0009(7) 0.0012(7)
C14 0.0152(9) 0.0076(8) 0.0099(8) -0.0017(6) -0.0004(7) 0.0034(7)
C15 0.0119(8) 0.0175(9) 0.0144(8) -0.0023(8) -0.0031(7) 0.0031(7)
C16 0.0142(9) 0.0150(9) 0.0122(8) -0.0033(7) 0.0017(7) -0.0024(8)
C17 0.0125(9) 0.0109(9) 0.0092(8) -0.0009(7) 0.0009(7) 0.0010(7)
C18 0.0121(9) 0.0111(9) 0.0103(8) -0.0015(7) 0.0021(7) 0.0000(7)
C19 0.0266(10) 0.0182(10) 0.0112(8) 0.0020(8) 0.0005(8) 0.0030(8)
C20 0.0162(9) 0.0176(10) 0.0118(8) -0.0001(7) 0.0015(8) 0.0036(8)
C21 0.0262(10) 0.0135(9) 0.0128(9) -0.0036(7) -0.0008(8) -0.0011(8)
C22 0.0223(10) 0.0139(10) 0.0135(9) -0.0012(7) 0.0036(8) -0.0025(8)
C23 0.0251(10) 0.0180(11) 0.0101(8) -0.0003(7) -0.0032(8) 0.0012(8)
C24 0.0124(8) 0.0179(10) 0.0100(8) -0.0027(8) 0.0000(8) 0.0014(7)
C25 0.0223(10) 0.0214(10) 0.0086(8) -0.0021(7) 0.0010(8) 0.0007(8)
C26 0.0302(11) 0.0222(10) 0.0113(8) 0.0030(8) 0.0004(8) -0.0004(9)
C27 0.0296(11) 0.0215(10) 0.0159(9) 0.0068(8) -0.0034(9) 0.0026(9)
C28 0.0228(10) 0.0143(9) 0.0173(9) 0.0025(7) -0.0011(8) 0.0046(8)
C29 0.0444(14) 0.0273(12) 0.0256(11) 0.0131(9) -0.0022(10) 0.0067(11)
C30 0.0308(12) 0.0396(13) 0.0179(9) 0.0064(9) -0.0081(9) 0.0014(11)

_geom_special_details
;
All esds (except the esd in the dihedral angle between two l.s. planes)
are estimated using the full covariance matrix. The cell esds are taken
into account individually in the estimation of esds in distances, angles
and torsion angles; correlations between esds in cell parameters are only
used when they are defined by crystal symmetry. An approximate (isotropic)
treatment of cell esds is used for estimating esds involving l.s. planes.
;

loop_
_geom_bond_atom_site_label_1
_geom_bond_atom_site_label_2
_geom_bond_distance
_geom_bond_site_symmetry_2
_geom_bond_publ_flag
Br15 C15 1.9789(18) . ?
O2 C02 1.423(2) . ?
O3 C03 1.4354(19) . ?
O14 C24 1.363(2) . ?
O14 C14 1.507(2) . ?
O23 C23 1.425(2) . ?
O24 C24 1.200(2) . ?
C01 C02 1.519(2) . ?
C01 C18 1.542(2) . ?
C02 C03 1.521(3) . ?
C03 C04 1.540(3) . ?

C04 C23 1.541(3) . ?
C04 C19 1.544(2) . ?
C04 C05 1.558(2) . ?
C05 C06 1.531(3) . ?
C05 C18 1.552(2) . ?
C06 C07 1.529(2) . ?
C07 C08 1.545(2) . ?
C08 C20 1.548(3) . ?
C08 C17 1.559(2) . ?
C08 C09 1.612(2) . ?
C09 C21 1.543(2) . ?
C09 C10 1.551(2) . ?
C09 C14 1.566(2) . ?
C10 C11 1.542(2) . ?
C11 C12 1.546(2) . ?
C12 C24 1.503(3) . ?
C12 C25 1.526(2) . ?
C12 C13 1.534(3) . ?
C13 C28 1.534(3) . ?
C13 C14 1.569(2) . ?
C14 C15 1.520(3) . ?
C15 C16 1.515(2) . ?
C16 C17 1.533(2) . ?
C17 C18 1.569(2) . ?
C18 C22 1.551(3) . ?
C25 C26 1.528(3) . ?
C26 C27 1.539(3) . ?
C27 C30 1.532(3) . ?
C27 C29 1.533(3) . ?
C27 C28 1.554(3) . ?

loop_
_geom_angle_atom_site_label_1
_geom_angle_atom_site_label_2
_geom_angle_atom_site_label_3
_geom_angle
_geom_angle_site_symmetry_1
_geom_angle_site_symmetry_3
_geom_angle_publ_flag
C24 O14 C14 110.61(13) . . ?
C02 C01 C18 113.27(15) . . ?
O2 C02 C01 107.06(15) . . ?
O2 C02 C03 111.10(14) . . ?
C01 C02 C03 112.11(15) . . ?
O3 C03 C02 106.72(14) . . ?
O3 C03 C04 112.12(14) . . ?

C02 C03 C04 112.46(14) . . ?
C03 C04 C23 108.63(15) . . ?
C03 C04 C19 111.40(15) . . ?
C23 C04 C19 103.95(15) . . ?
C03 C04 C05 106.47(14) . . ?
C23 C04 C05 109.87(15) . . ?
C19 C04 C05 116.36(15) . . ?
C06 C05 C18 111.70(14) . . ?
C06 C05 C04 115.09(15) . . ?
C18 C05 C04 115.77(15) . . ?
C07 C06 C05 109.66(15) . . ?
C06 C07 C08 113.02(15) . . ?
C07 C08 C20 105.78(15) . . ?
C07 C08 C17 109.02(14) . . ?
C20 C08 C17 112.15(15) . . ?
C07 C08 C09 109.53(14) . . ?
C20 C08 C09 112.96(14) . . ?
C17 C08 C09 107.35(13) . . ?
C21 C09 C10 107.86(15) . . ?
C21 C09 C14 109.53(15) . . ?
C10 C09 C14 107.06(14) . . ?
C21 C09 C08 110.04(15) . . ?
C10 C09 C08 110.96(14) . . ?
C14 C09 C08 111.29(14) . . ?
C11 C10 C09 114.10(14) . . ?
C10 C11 C12 112.68(14) . . ?
C24 C12 C25 114.50(16) . . ?
C24 C12 C13 99.91(14) . . ?
C25 C12 C13 111.72(15) . . ?
C24 C12 C11 106.66(14) . . ?
C25 C12 C11 113.90(15) . . ?
C13 C12 C11 109.12(14) . . ?
C28 C13 C12 111.01(15) . . ?
C28 C13 C14 127.56(15) . . ?
C12 C13 C14 99.80(14) . . ?
O14 C14 C15 100.99(14) . . ?
O14 C14 C09 105.47(13) . . ?
C15 C14 C09 116.96(14) . . ?
O14 C14 C13 97.77(13) . . ?
C15 C14 C13 114.38(15) . . ?
C09 C14 C13 117.11(15) . . ?
C16 C15 C14 114.45(15) . . ?
C16 C15 Br15 108.51(12) . . ?
C14 C15 Br15 112.03(12) . . ?
C15 C16 C17 111.76(15) . . ?
C16 C17 C08 109.63(14) . . ?

C16 C17 C18 112.61(15) . . ?
C08 C17 C18 117.79(14) . . ?
C01 C18 C22 108.77(15) . . ?
C01 C18 C05 106.79(14) . . ?
C22 C18 C05 113.80(15) . . ?
C01 C18 C17 107.11(14) . . ?
C22 C18 C17 111.86(14) . . ?
C05 C18 C17 108.19(14) . . ?
O23 C23 C04 114.81(15) . . ?
O24 C24 O14 120.55(17) . . ?
O24 C24 C12 130.81(17) . . ?
O14 C24 C12 108.61(14) . . ?
C12 C25 C26 110.67(15) . . ?
C25 C26 C27 113.95(17) . . ?
C30 C27 C29 108.32(18) . . ?
C30 C27 C26 111.09(17) . . ?
C29 C27 C26 108.83(18) . . ?
C30 C27 C28 111.45(18) . . ?
C29 C27 C28 107.68(17) . . ?
C26 C27 C28 109.37(16) . . ?
C13 C28 C27 110.91(16) . . ?

loop_
_geom_torsion_atom_site_label_1
_geom_torsion_atom_site_label_2
_geom_torsion_atom_site_label_3
_geom_torsion_atom_site_label_4
_geom_torsion
_geom_torsion_site_symmetry_1
_geom_torsion_site_symmetry_2
_geom_torsion_site_symmetry_3
_geom_torsion_site_symmetry_4
_geom_torsion_publ_flag
C18 C01 C02 O2 -177.78(14) . . . . ?
C18 C01 C02 C03 -55.7(2) . . . . ?
O2 C02 C03 O3 -60.15(18) . . . . ?
C01 C02 C03 O3 -179.89(15) . . . . ?
O2 C02 C03 C04 176.52(14) . . . . ?
C01 C02 C03 C04 56.77(19) . . . . ?
O3 C03 C04 C23 66.31(19) . . . . ?
C02 C03 C04 C23 -173.42(14) . . . . ?
O3 C03 C04 C19 -47.6(2) . . . . ?
C02 C03 C04 C19 72.66(18) . . . . ?
O3 C03 C04 C05 -175.41(14) . . . . ?
C02 C03 C04 C05 -55.15(18) . . . . ?
C03 C04 C05 C06 -169.77(15) . . . . ?

C23 C04 C05 C06 -52.3(2) . . . . ?
C19 C04 C05 C06 65.4(2) . . . . ?
C03 C04 C05 C18 57.43(19) . . . . ?
C23 C04 C05 C18 174.88(15) . . . . ?
C19 C04 C05 C18 -67.4(2) . . . . ?
C18 C05 C06 C07 -62.30(19) . . . . ?
C04 C05 C06 C07 163.03(15) . . . . ?
C05 C06 C07 C08 60.7(2) . . . . ?
C06 C07 C08 C20 69.44(19) . . . . ?
C06 C07 C08 C17 -51.3(2) . . . . ?
C06 C07 C08 C09 -168.53(15) . . . . ?
C07 C08 C09 C21 49.5(2) . . . . ?
C20 C08 C09 C21 167.16(16) . . . . ?
C17 C08 C09 C21 -68.69(18) . . . . ?
C07 C08 C09 C10 -69.75(18) . . . . ?
C20 C08 C09 C10 47.9(2) . . . . ?
C17 C08 C09 C10 172.02(14) . . . . ?
C07 C08 C09 C14 171.15(14) . . . . ?
C20 C08 C09 C14 -71.23(19) . . . . ?
C17 C08 C09 C14 52.92(18) . . . . ?
C21 C09 C10 C11 75.62(19) . . . . ?
C14 C09 C10 C11 -42.17(19) . . . . ?
C08 C09 C10 C11 -163.79(15) . . . . ?
C09 C10 C11 C12 49.2(2) . . . . ?
C10 C11 C12 C24 43.39(19) . . . . ?
C10 C11 C12 C25 170.70(16) . . . . ?
C10 C11 C12 C13 -63.70(19) . . . . ?
C24 C12 C13 C28 178.41(14) . . . . ?
C25 C12 C13 C28 56.9(2) . . . . ?
C11 C12 C13 C28 -69.96(18) . . . . ?
C24 C12 C13 C14 -44.80(16) . . . . ?
C25 C12 C13 C14 -166.31(15) . . . . ?
C11 C12 C13 C14 66.84(17) . . . . ?
C24 O14 C14 C15 -145.67(13) . . . . ?
C24 O14 C14 C09 92.13(15) . . . . ?
C24 O14 C14 C13 -28.87(17) . . . . ?
C21 C09 C14 O14 -168.69(14) . . . . ?
C10 C09 C14 O14 -51.99(16) . . . . ?
C08 C09 C14 O14 69.41(17) . . . . ?
C21 C09 C14 C15 80.05(19) . . . . ?
C10 C09 C14 C15 -163.26(15) . . . . ?
C08 C09 C14 C15 -41.9(2) . . . . ?
C21 C09 C14 C13 -61.3(2) . . . . ?
C10 C09 C14 C13 55.42(19) . . . . ?
C08 C09 C14 C13 176.83(14) . . . . ?
C28 C13 C14 O14 170.68(17) . . . . ?

C12 C13 C14 O14 44.41(15) . . . . ?
C28 C13 C14 C15 -83.5(2) . . . . ?
C12 C13 C14 C15 150.26(15) . . . . ?
C28 C13 C14 C09 58.8(2) . . . . ?
C12 C13 C14 C09 -67.45(18) . . . . ?
O14 C14 C15 C16 -74.22(17) . . . . ?
C09 C14 C15 C16 39.6(2) . . . . ?
C13 C14 C15 C16 -178.06(15) . . . . ?
O14 C14 C15 Br15 161.71(10) . . . . ?
C09 C14 C15 Br15 -84.49(16) . . . . ?
C13 C14 C15 Br15 57.86(17) . . . . ?
C14 C15 C16 C17 -49.1(2) . . . . ?
Br15 C15 C16 C17 76.82(16) . . . . ?
C15 C16 C17 C08 63.17(19) . . . . ?
C15 C16 C17 C18 -163.60(15) . . . . ?
C07 C08 C17 C16 177.20(14) . . . . ?
C20 C08 C17 C16 60.41(18) . . . . ?
C09 C08 C17 C16 -64.23(18) . . . . ?
C07 C08 C17 C18 46.7(2) . . . . ?
C20 C08 C17 C18 -70.10(19) . . . . ?
C09 C08 C17 C18 165.26(14) . . . . ?
C02 C01 C18 C22 -70.13(19) . . . . ?
C02 C01 C18 C05 53.07(19) . . . . ?
C02 C01 C18 C17 168.80(15) . . . . ?
C06 C05 C18 C01 169.46(14) . . . . ?
C04 C05 C18 C01 -56.19(19) . . . . ?
C06 C05 C18 C22 -70.51(19) . . . . ?
C04 C05 C18 C22 63.8(2) . . . . ?
C06 C05 C18 C17 54.47(18) . . . . ?
C04 C05 C18 C17 -171.19(14) . . . . ?
C16 C17 C18 C01 67.71(18) . . . . ?
C08 C17 C18 C01 -163.17(15) . . . . ?
C16 C17 C18 C22 -51.4(2) . . . . ?
C08 C17 C18 C22 77.7(2) . . . . ?
C16 C17 C18 C05 -177.51(14) . . . . ?
C08 C17 C18 C05 -48.4(2) . . . . ?
C03 C04 C23 O23 70.32(19) . . . . ?
C19 C04 C23 O23 -170.96(15) . . . . ?
C05 C04 C23 O23 -45.8(2) . . . . ?
C14 O14 C24 O24 -177.62(16) . . . . ?
C14 O14 C24 C12 0.79(18) . . . . ?
C25 C12 C24 O24 -33.8(3) . . . . ?
C13 C12 C24 O24 -153.28(19) . . . . ?
C11 C12 C24 O24 93.2(2) . . . . ?
C25 C12 C24 O14 148.03(15) . . . . ?
C13 C12 C24 O14 28.53(17) . . . . ?

C11 C12 C24 O14 -85.01(17) . . . . ?
C24 C12 C25 C26 -166.60(16) . . . . ?
C13 C12 C25 C26 -53.9(2) . . . . ?
C11 C12 C25 C26 70.3(2) . . . . ?
C12 C25 C26 C27 53.8(2) . . . . ?
C25 C26 C27 C30 69.2(2) . . . . ?
C25 C26 C27 C29 -171.60(16) . . . . ?
C25 C26 C27 C28 -54.2(2) . . . . ?
C12 C13 C28 C27 -57.6(2) . . . . ?
C14 C13 C28 C27 -179.24(18) . . . . ?
C30 C27 C28 C13 -68.0(2) . . . . ?
C29 C27 C28 C13 173.36(18) . . . . ?
C26 C27 C28 C13 55.3(2) . . . . ?

_diffrn_measured_fraction_theta_max 0.990
_diffrn_reflns_theta_full 64.14
_diffrn_measured_fraction_theta_full 0.990
_refine_diff_density_max 0.329
_refine_diff_density_min -0.412
_refine_diff_density_rms 0.080
